# Dynamic and Reversible Tuning of Hydrogel Viscoelasticity by Transient Polymer Interactions for Controlling Cell Adhesion

**DOI:** 10.1002/adma.202408616

**Published:** 2025-02-11

**Authors:** Shane Scott, Maria Villiou, Federico Colombo, Angeles De la Cruz‐García, Leon Tydecks, Lotta Toelke, Katharina Siemsen, Christine Selhuber‐Unkel

**Affiliations:** ^1^ Department of Materials Science and Engineering McMaster University 1280 Main St. W. Hamilton Ontario L8S 4L8 Canada; ^2^ Institute for Molecular Systems Engineering and Advanced Materials (IMSEAM) Heidelberg University D‐69120 Heidelberg Germany; ^3^ Max Planck School Matter to Life Heidelberg University Jahnstraße 29 69120 Heidelberg Germany; ^4^ Max Planck Institute for Polymer Research Ackermannweg 10A 55128 Mainz Germany; ^5^ Institute for Materials Science Kiel University Kaiserstraße 2 24143 Kiel Germany

**Keywords:** controlled cell culture, dynamic cell culture, hydrogels, hydrogen bonding, reversible viscoelastic properties

## Abstract

Cells are highly responsive to changes in their mechanical environment, influencing processes such as stem cell differentiation and tumor progression. To meet the growing demand for materials used for high throughput mechanotransduction studies, simple means of dynamically adjusting the environmental viscoelasticity of cell cultures are needed. Here, a novel method is presented to dynamically and reversibly control the viscoelasticity of naturally derived polymer hydrogels through interactions with poly (ethylene glycol) (PEG). Interactions between PEG and hydrogel polymers, possibly involving hydrogen bonding, stiffen the hydrogel matrices. By dynamically changing the PEG concentration of the solution in which polymer hydrogels are incubated, their viscoelastic properties are adjusted, which in turn affects cell adhesion and cytoskeletal organization. Importantly, this effects is reversible, providing a cost‐effective and simple strategy for dynamically adjusting the viscoelasticity of polymer hydrogels. This method holds promise for applications in mechanobiology, biomedicine, and the life sciences.

## Graphical abstract

Biological systems are very responsive to changes in stiffness, influencing important processes, and diseases in humans. Here, a novel method to dynamically and reversibly control hydrogel stiffness through interactions with poly (ethylene glycol) (PEG) is presented. By exposing hydrogels to PEG solutions at different concentrations and sizes, hydrogel stiffness can be tuned, in turn affecting cell properties.

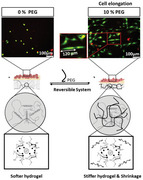

## 1 Introduction

Mechanical signals from the extracellular environment have a significant impact on cell fate.^[^ ^1^, ^2^, ^3^ ^]^ They can direct stem cell differentiation,^[^ ^4^, ^5^, ^6^ ^]^ cell proliferation,^[^ ^7^, ^8^ ^]^ tumor progression,^[^ ^9^ ^]^ and remodelling of the cytoskeleton.^[^ ^10^ ^]^ Cytoskeletal elements and focal adhesions are strongly involved in such mechanosensory processes.^[^ ^11^ ^]^ In vivo, cells can be exposed to a wide range of mechanical environments over time, with possible implications for cell function. Cells themselves create the extracellular matrix environment during tissue development and dynamically change it over time as a function of changes in the surrounding environment,^[^ ^12^ ^]^ though not typically enough to alter the surrounding stiffness of synthetic cell culture scaffolds.^[^ ^13^ ^]^ The development of a technique that can easily and reversibly modulate the cellular environment to any mechanical state is crucial for studying how mechanical influences affect cells, and understanding the complexities of mechanotransduction and extracellular matrix dynamics. Furthermore, the invention of a method that could be applied to any kind of hydrogel would allow researchers the flexibility to choose the optimal cellular scaffold for their individual experiments.

Alginate hydrogels, which mimic the biocompatible extracellular matrix,^[^ ^14^ ^]^ are easy to handle and have applications in drug delivery,^[^ ^15^, ^16^ ^]^ wound dressing,^[^ ^16^, ^17^ ^]^ and as cellular scaffolds.^[^ ^18^, ^19^ ^]^ Chemically modifying alginate polymers instead of physically crosslinking them permits fine control of the cell scaffold system, giving the ability to regulate adhesion sites and hydrogel stiffness.^[^ ^20^ ^]^ Even viscosity can be tuned independently from elasticity by covalently bonding poly (ethylene glycol) (PEG) molecules of different lengths to the polymer network, causing stress energy to be dissipated by the freely‐hanging PEG molecules and subsequently allowing for stress relaxation time tuning.^[^ ^21^ ^]^ However, existing methods rely on irreversible chemical modifications, and dynamically adjusting the mechanical properties of alginate hydrogels remains a challenge for biomedical applications.^[^ ^22^ ^]^

To make use of mechanotransduction in triggering cells for large‐scale and biotechnological applications, there is a strong demand for simple methods that dynamically tune the mechanics of cellular environments in a reversible way that is easily upscaled. Several materials have been introduced that allow for control of extracellular environments’ mechanical properties. For example, the integration of light‐switchable molecules into poly (ethylene‐glycol)‐based hydrogels permits control of their mechanical properties due to a conformational change in the photoswitches.^[^ ^23^, ^24^ ^]^ Similarly, the incorporation of photodegradable molecules in hydrogels dynamically tunes a continuous range of substrate stiffness.^[^ ^25^, ^26^ ^]^ Another strategy uses host‐guest interactions with solutes that physically crosslink molecules incorporated in the hydrogel, changing their stiffness.^[^ ^27^, ^28^ ^]^ The creation of these photoswitchable or physically crosslinked hydrogels can require complex chemistry involving several steps, and can sometimes be difficult to integrate into hydrogels.

**Figure 1:**
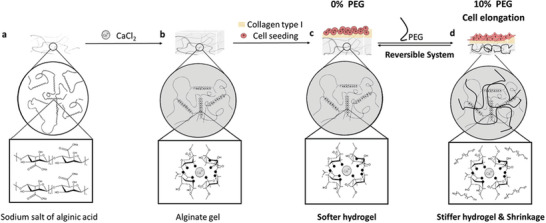
Overview: Principle – Dynamic PEG‐Hydrogel Interactions. Schematics of PEG‐mediated dynamic viscoelastic control of alginate gels affecting cell morphology. Alginate structure of a) non‐modified hydrogels (gray strands), b) CaCl_2_ cross‐linked alginate polymers (gray blocks). c) Surface functionalization of alginate hydrogels with collagen type I (yellowish) support cell attachment and adhesion. d) Hydrogel pores permit PEG polymers (black lines) to both enter the hydrogel, allowing for PEG‐hydrogel interactions, and for solvent molecules to exit through diffusion. Cell attachment to the alginate was controlled by modifying PEG concentration: from 0% PEG to 10% PEG (10 days with PEG) and back to 0% PEG (10 days without PEG); and vice versa (c,d).

In this work, we use PEG molecules to tune the mechanical properties of alginate hydrogels dynamically and reversibly during cell culture (**Figure** **1**). PEG is capable of intramolecular and intermolecular hydrogen bonding through their hydroxyl groups and ether oxygens.^[^ ^29^, ^30^ ^]^ Hydrogen bonding has been previously used to crosslink hydrogels,^[^ ^31^ ^]^ and increasing hydrogen bonding in PEG‐based hydrogels has shown increased stiffness.^[^ ^32^ ^]^ We assume that adding polymers capable of hydrogen bonding to a hydrogel's polymers will cause it to stiffen. Since the added PEG molecules are not covalently bonded, they are simple to remove. PEG is known to be biocompatible and have little effect on cell adhesion.^[^ ^33^ ^]^ By changing the PEG concentration of cell culture media, polymer hydrogel mechanical properties are tuned over a period of days. Additionally, while PEG is used in this work, the technique could theoretically be extended to use nearly any polymer capable of interacting with it.

## 2 Results and Discussion

### 2.1 Dynamic Interactions in Hydrogels

In this work, we use alginate hydrogels as polymer substrates for cell culture due to their biocompatibility, low cost, low toxicity, and high accessibility.^[^ ^14^ ^]^ Furthermore, alginate molecules are also capable of hydrogen bonding via their hydroxyl and carboxyl groups,^[^ ^34^ ^]^ potentially allowing them to interact with PEG molecules via hydrogen bonding.

We postulate that PEG dissolved in a solvent can penetrate hydrogels incubated in it, hydrogen bonding with the hydrogel polymers capable of such cross‐linking. The relation between a hydrogel network's mechanical properties and crosslinking is

(1) $$G_{0}=\frac{4}{5}\frac{\rho \phi RT}{M_{c}}$$

where ρ is the polymer network density, ϕ is the polymer volume fraction, *R* is the ideal gas constant, *T* is the temperature, and *M_c_* is the molecular weight of the polymer strand between successive crosslinks or entanglements.^[^ ^35^ ^]^

**Figure 2:**
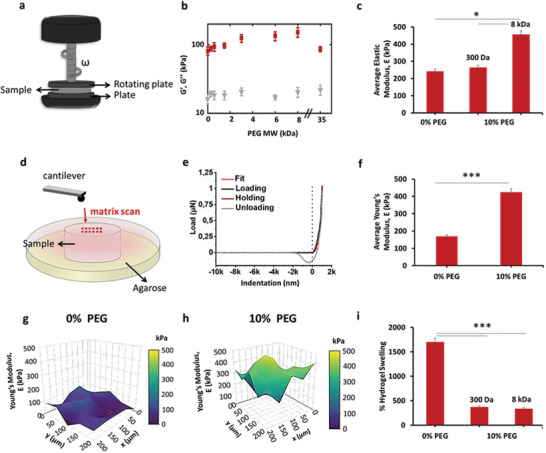
Mechanical characterization of 2% (w/v) alginate without and with 10% 8 kDa (or 300 Da) PEG incubation. a) Schematic representation of oscillatory rheology. b) Effect of PEG molecular weight (MW) on storage modulus *G*′ and loss modulus *G*″ for 2% (w/v) alginate hydrogels with/without 10% (w/v) PEG (300 to 35 000 Da), as measured by oscillatory rheology at pH 7.5, 1 Hz frequency, 0.1% shear strain and 37 °C. c) Effect of incubating 2% alginate hydrogels with/without 10% (w/v) 300 Da or 8 kDa PEG on elastic modulus *E*. Number of gels for each condition: 8. d) Schematic of the microindentation experiment. e) Representative curve of a microindentation measurement. f) Average Young's Modulus 〈*E*〉 measured by fitting the microindentation data, for hydrogel samples with/without 10% (w/v) 8 kDa PEG. Number of gels for each condition: 3, number of matrix scans: 3 per gel, number of curves per matrix scan: 25. Measurements of *E* at 25 different positions (x, y) for 2% alginate hydrogels either g) without, or h) with 10% (w/v) 8 kDa PEG. i) Swelling percentage of 2% (w/v) alginate hydrogels with/without 10% (w/v) PEG (300 Da and 8 kDa). Number of hydrogels for each condition: 5. Data are presented as the mean ± standard deviation and analysed using the one‐way Analysis of Variance (ANOVA) with ^*^ *p* < 0.05 and ^***^ *p* < 0.001.

To characterize the viscoelastic properties of the hydrogel, the dynamic shear modulus G* = *G*′  + i*G*″ (*G*′: storage modulus; *G*″: loss modulus) is measured by applying an oscillating stress (or strain) to the hydrogel through a rheometer in a plate‐plate geometry (**Figure** **2**a; see Section S1, Supporting Information). However, when these hydrogels are transferred to a PEG‐containing solution of any molecular weight, PEG molecules diffuse into the hydrogel, interacting with the polymers composing the alginate hydrogel network, increasing the hydrogel stiffness (Figure 2b,c). Interestingly, *G*″ values (Figure 2b) remained relatively constant regardless of the presence of different PEG molecule sizes. Molecular interactions with the network polymers by penetrating PEG molecules could explain small changes in *G*″: PEG molecules hydrogen bonded with alginate polymers are pulled when the hydrogel is deformed, dissipating energy and increasing *G*″. See Section S1 and Figure S11 (Supporting information) for more details.

With increasing PEG MW, *G*′ steadily increases until a maximum is reached at 8 kDa, with a significant decrease measured at 35 kDa (Figure 2b). The steady increase of *G*′ with PEG MW from 300 Da to 8 kDa could be due to several possibilities: crowding, solvent swelling (affecting hydrogel concentration), polymer phase separation, polymer entanglements, or hydrogen bonding. Crowding‐induced stiffness occurs when crowding molecules (PEG, in this case) take up space in the hydrogel polymer network, reducing the possible structures that the hydrogel polymers could dynamically adopt, in turn stiffening the network by reducing the system entropy. If crowding were the main factor affecting the network modulus, *G*′ would be independent of PEG MW as we used the same mass concentration of PEG in solution, 10% (w/v), for all experiments, thereby crowding all hydrogels by similar amounts. However, *G*′ increases with PEG MW despite equal amounts of crowding, indicating that crowding is not the main factor controlling changes in hydrogel stiffness. Additionally, in previous work, crowding had little effect on the morphology of rat embryonic fibroblasts,^[^ ^36^ ^]^ indicating that it cannot explain the stretched morphologies observed here.

To determine whether solvent swelling (which changes hydrogel concentration) was the main contributor to *G*′, we measured the hydrogel swelling percentage via gravimetric analysis (Figure 2i). In these experiments, the mass of hydrogels with solution is compared to their mass once completely dried. Only small differences in swelling percentage were observed for hydrogels incubated with either 300 or 8 kDa PEG, indicating that similar amounts of water were absorbed by each before drying, and were thus at similar hydrogel polymer concentrations. As the swelling percentage for these hydrogels was very similar, indicating nearly identical concentrations despite vast differences in *G*′, solvent‐induced changes in hydrogel concentration cannot account for changes in stiffness. If increased hydrogel concentration due to water diffusion out of the hydrogel was responsible for the increase in *G*′, then both 300 Da and 8 kDa PEG‐incubated hydrogels would have the same stiffness as their swelling percentages were nearly identical. As this is not the case, solvent‐swelling‐induced changes in hydrogel concentration cannot explain the observed changes in hydrogel stiffness.

While polymer‐driven phase separation could also explain the change in stiffness, PEG mixed with alginate forms a solution, as demonstrated by Liu, et al.,^[^ ^37^ ^]^ and thus would not phase separate. As to entanglements, topological entanglements cannot occur in this system as the sizes of the PEG polymers are too small compared to the pore size of the alginate hydrogel. Polymers of 8 kDa PEG have *R_G_* ≅ 3.9 nm (in line with experimental measurements of PEG size),^[^ ^38^, ^39^ ^]^ smaller than the alginate pore size (5 nm for 2% (w/v) alginate).^[^ ^40^ ^]^ For topological entanglements to occur, the PEG length would have to be sufficiently long to reach at least two separate alginate polymers, which is not the case. While cohesion entanglements could occur in this system, the Van der Waals forces associated with this mechanism are too weak to explain the strong increase in hydrogel stiffness observed. Thus, topological and cohesion entanglements are unlikely, leaving hydrogen bonding as the most likely mechanism by which these hydrogels are stiffening.

Since PEG and hydrogel polymers in general can hydrogen bond, we postulate that PEG‐induced increases in hydrogel *G*′ are caused by hydrogen bonding between PEG and the hydrogel polymers. Hydrogen bonds are weaker than covalent bonds, allowing for the observed dynamic changes in *G*′ as they can bind and unbind at physiological temperatures while being simultaneously strong enough to explain the large increases in hydrogel stiffness. Finally, longer PEG molecules can create more hydrogen bonds, allowing for more crosslinking with hydrogel polymers.

PEG‐induced increases in *E* between the native alginate hydrogel and that of one incubated with 8 kDa PEG are fairly significant, with an increase of over 200 kPa (more than double, Figure 2c). This increase in mechanical properties is of a similar order of magnitude as that occurs when heating thermoresponsive materials,^[^ ^41^ ^]^ and that of photoactivated hydrogels integrated with either photoswitchable or photodegradable molecules.^[^ ^23^, ^25^ ^]^ Additionally, when considering the range of *G*′ values accessed using various PEG MW (Figure 2b), this PEG‐interaction technique demonstrates a range of available control for viscoelastic tuning of polymer hydrogels. Furthermore, no complex chemistry or modifications of the polymer hydrogels were required when using PEG interactions, as is the case with hydrogels modified to integrate either photoswitchable molecules,^[^ ^23^, ^24^ ^]^ photodegradable molecules,^[^ ^25^, ^26^ ^]^ or physical (host) crosslinking sites.^[^ ^27^, ^28^ ^]^ Finally, no change in temperature is necessary for these PEG interactions, an advantage for cell culture experiments when considering thermoresponsive polymer cell scaffolds as thermal stability can be maintained.

The decrease in *G*′ at 35 kDa PEG MW compared to that at 8 kDa is likely due to the difference in size of the 35 kDa PEG and the alginate pores. As the 35 kDa PEG molecules are bigger than the pore size, the likelihood of them entering the hydrogel compared to the 8 kDa PEG is much smaller, reducing the interactions between 35 kDa PEG and alginate polymers, and thus *G*′.

To investigate local variations in hydrogel stiffness, microindentation measurements of Young's modulus (*E*′) on hydrogel surfaces were performed (Figure 2d–h). The average of these local measurements of surface elasticity (Figure 2f) were within a similar range to bulk rheometry measurements (Figure 2c).

### 2.2 PEG Diffusion into Alginate Hydrogels

**Figure 3:**
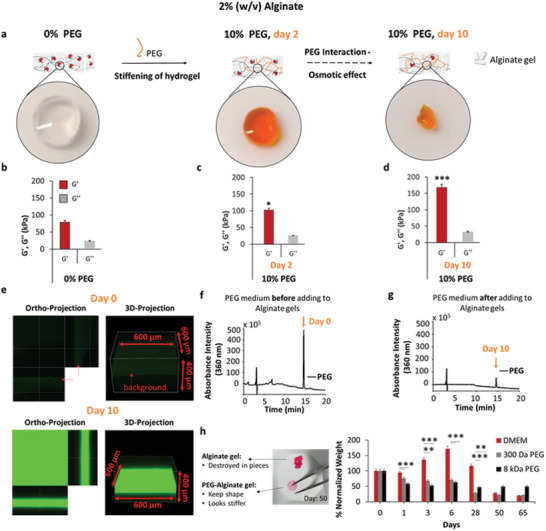
Visualization of PEG diffusion on alginate gels. a) Schematics of 2% alginate hydrogels incubated with fluorescent 10% (w/v) 8 kDa FITC‐PEG after 0 days, 2 days, and 10 days. b–d) Oscillatory rheometry measurements of *G*′ and *G*″ for 2% alginate hydrogels incubated in 10% (w/v) FITC‐PEG for (b) 0 days, (c) 2 days, and (d) 10 days, at pH 7.5, 1 Hz frequency, 0.1% shear strain, and 37 °C. e) Ortho‐projection and 3D projection of alginate hydrogels without PEG (day 0) or with 10% (w/v) 8 kDa FITC‐PEG (day 10), imaged using confocal microscopy. RP‐HPLC analysis of the absorbance intensity at 360 nm of the FITC‐PEG medium: f) before incubation with alginate hydrogels, and g) after incubation. h) Hydrolytic stability of the alginate and PEG‐alginate hydrogels was determined by a gravimetric method and measured over time. The hydrogels were swollen and stored in the incubator at 37 °C in DMEM‐ 25 mm HEPES buffer, pH 7.5 for 65 days. At each time point, hydrogel mass was measured. Number of hydrogels for each condition: 5. Data are presented as the mean ± standard deviation and analysed using the one‐way Analysis of Variance (ANOVA) with ^*^ *p* < 0.05, ^**^ *p* < 0.01, and ^***^ *p* < 0.001.

We subsequently observed the changes in hydrogel mechanics as a function of time, as shown in **Figure** **3**. After incubation in a solution containing fluorescent PEG (FITC‐PEG), hydrogels clearly shrunk, with the orange color indicating the presence of penetrating FITC‐PEG (Figure 3a). Over the course of 10 days, *G*′ more than doubled, with little change in *G*″ (Figure 3b–d). This change of *G*′ is in a similar range as achieved by other dynamic, though more complicated, techniques.^[^ ^23^, ^25^, ^27^ ^]^ To quantify fluorescent PEG uptake, we imaged samples of alginate hydrogels incubated with/without FITC‐PEG (Figure 3e). Simultaneously, absorbance measurements of FITC‐PEG solutions before incubation (Figure 3f) and after 10 days of incubation (Figure 3g) were performed using RP‐HPLC. A more than fivefold decrease in absorbance intensity at 360 nm was observed, providing additional evidence of FITC‐PEG incorporation into the hydrogel. RP‐HPLC analysis confirmed 30–40% consumption of the initial PEG solution after 10 days of incubation (Figure S24, Supporting information).

As alginate degrades over time,^[^ ^42^ ^]^ hydrolytic stability studies were performed for 2% (w/v) alginate hydrogels incubated in Dulbecco's Modified Eagle Medium (DMEM) for a week at 4 °C without PEG, with 10% (w/v) 300 Da PEG, or with 8 kDa PEG. In the latter hydrogels, ample time was given to permit PEG to diffuse into the hydrogels. All hydrogels were kept in their respective media for 65 days at 37 °C to mimic long‐term cellular conditions and their weights were measured gravimetrically (Figure 3h). In the first week, alginate hydrogels not exposed to PEG absorbed media, while those incubated in PEG did not, likely due to osmotic pressure driving solvent out of the hydrogels and into PEG‐filled solution. Thus, PEG‐exposed alginate hydrogels likely had a lower water content than those exposed purely to DMEM, which may increase cell adhesion for cells grown on alginate hydrogels not exposed to PEG.^[^ ^43^ ^]^ For alginate hydrogels incubated in PEG media, a steadier mass decrease was observed, with greater losses occurring for hydrogels incubated with 300 Da PEG, perhaps due to less crosslinking via hydrogen bonding. The weight stabilization of alginate hydrogels incubated with 8 kDa PEG after 65 days compared to other samples indicates that PEG interactions also protect polymer hydrogels against degradation. This is particularly useful in long‐term cell studies.

### 2.3 Stiffness Range and Timescales of Polymer Hydrogel & PEG Interactions

**Figure 4:**
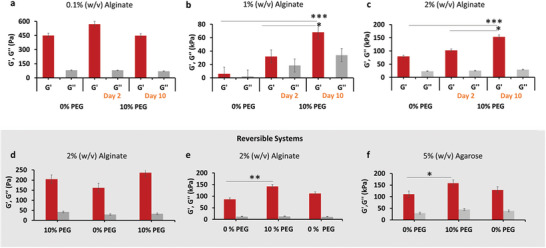
PEG‐interaction effect on alginate or agarose hydrogels, for 10% (w/v) 8 kDa PEG on storage (*G*′,  *red* *color*) and loss (*G*″,  *gray* *color*) moduli, as measured by oscillatory rheology at constant pH 7.5, 1 Hz frequency, 0.1% shear strain, and 37 °C. Interaction effects on 0.1% a), 1% b), and 2% c) alginate hydrogels. Number of hydrogels for each condition: 8. d,e) Effect of dynamically changing PEG concentration every 10 days on storage (*G*′,  *red* *color*) and loss (*G*″,  *gray* *color*) moduli for 2% alginate hydrogels, as measured by oscillatory rheology at constant pH 7.5, 1 Hz frequency, 0.1% shear strain and 37 °C. Number of hydrogels for each condition: 5. Data are presented as the mean ± standard deviation and analysed using the one‐way Analysis of Variance (ANOVA) with ^*^ *p* < 0.05, ^**^ *p* < 0.01, and ^***^ *p* < 0.001.

To determine the range and timescales of PEG interactions effects on polymer hydrogels, various samples using different alginate concentrations were made. Viscoelastic properties (*G*′ and *G*″) of 0.1%, 1%, and 2% (w/v) alginate hydrogels were measured without PEG present. They were then placed in a 10% (w/v) 8 kDa PEG solution and left for an additional 2–10 days, when *G*′ and *G*″ were measured (**Figure** **4**). For less concentrated alginate hydrogels, less PEG interactions will occur as there are less alginate polymers that can interact with PEG than at higher alginate concentrations, causing only minor changes in *G*′ and *G*″ (Figure 4a). For higher alginate concentrations, more alginate polymers offer easier access for PEG to interact, increasing the overall stiffness of the materials when PEG is introduced (Figure 4b,c). While the hydrogel samples in these experiments were incubated for a total of 10 days to increase stiffness, *G*′ increased after 2 days for alginate concentrations of 1% or more, with 1% alginate showing an almost tenfold increase (Figure 4b). This rapid, dynamic change in *G*′ shows that only short incubation times in PEG solutions allow an increase in polymer hydrogel stiffness. If this process is reversed by putting an alginate hydrogel previously exposed to PEG in a bath with no PEG, hydrogel mechanical stiffness decreases (Figure 4d). Hydrogel *G*′ can be increased yet again by incubation in a PEG‐containing solution. While the speed at which these reversible changes occur is not as fast as the minutes‐ to hours‐long timescales of hydrogels integrated with photoswitchable molecules^[^ ^23^ ^]^ or physical crosslinking interaction sites,^[^ ^27^ ^]^ it is sufficiently fast to affect hydrogel stiffness at time‐scales of long‐term cell culture. Additionally, the reversibility of this technique can be used for different hydrogels capable of hydrogen bonding(Figure 4e), such as agarose which contains ether and hydroxyl groups.^[^ ^44^ ^]^ When we incubated agarose hydrogels in solutions containing 10% (w/v) 8 kDa PEG, stiffening is also observed (Section S12, Supporting information) indicating that PEG hydrogen bonding can stiffen polymer hydrogels other than alginate. This offers an additional advantage over unidirectional techniques, such as photodegradable hydrogels whose mechanical properties cannot return to previous values once dynamically changed.^[^ ^25^, ^26^ ^]^ Furthermore, due to the long timescales over which these forces are dynamically applied, this PEG interaction technique may provide insight into the cellular tuning of the extracellular matrix.

### 2.4 Control of Cell Attachment, Growth, and Morphology by Dynamic PEG Concentration Tuning

**Figure 5:**
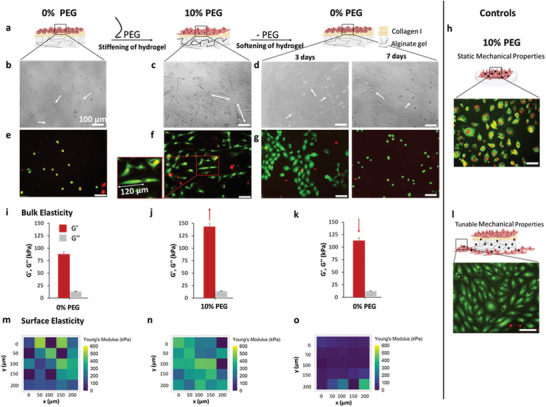
Control of cell adhesion by dynamic PEG concentration tuning. 2D cell culture of REF52 WT fibroblast performed on collagen type I functionalized 2% (w/v) alginate hydrogels, using 10% (w/v) 8 kDa PEG to dynamically tune the materials properties. a) Schematics of the PEG interaction‐mediated control of cell expansion on the matrixes. b–g) Cell adhesion to the alginate was controlled by modifying PEG concentration: from 0% PEG to 10% PEG (10 days with PEG) and back to 0% PEG (3 days and 7 days without PEG). Cell morphology changes from globular (b,e) to stretched (c,f) and back to globular (d,g) as hydrogel viscoelasticity changes. A cell viability of >95% was observed with a live/dead assay (live cells are stained green, while dead appeared in red), indicating good cytocompatibility for PEG‐interacting alginate hydrogels. h) Internal control: cells grown on a plastic well plate and incubated in 10% (w/v) 8 kDa PEG with no alginate hydrogel in the well. i–k) The effect of changing PEG concentration (every 10 days) on *G*′ and *G*″ for alginate hydrogels, as measured by oscillatory rheology at pH 7.5, 1 Hz frequency, 0.1% shear strain, and 37 °C. l) Internal control: cells grown on a plastic well plate beside 2% alginate hydrogels and incubated in 10% 8 kDa PEG. m–o) Effect of changing PEG concentration every 10 days on alginate hydrogel *E*, measured by matrix scan microindentation. Scale bars: 100 µm, number of independent experiments: 3, number of technical replicates per experiment: 3, number of images from which the shown representative images were chosen: 3. Data are presented as the mean ± standard deviation.

The effect of dynamic alginate hydrogel stiffness adjustment on cells was tested by dynamically changing the PEG concentration in cell media from 0% to 10%, then back to 0% (**Figure** **5**a–g). REF52 WT fibroblasts were grown on surface functionalized alginate hydrogels with collagen type I, used as cell adhesion points.^[^ ^45^, ^46^ ^]^ Collagen has long been used as a cell anchoring protein,^[^ ^45^ ^]^ especially for REF 52 cells,^[^ ^47^ ^]^ and previous work has shown that such collagen coatings do not affect hydrogel stiffness.^[^ ^48^ ^]^ The fibroblast samples were imaged under a microscope using a live/dead assay (Figure 5e–l). Before incubation in PEG solution, cells demonstrated a round morphology (Figure 5b,e). After 10 days of PEG incubation, *G*′ and *E*′ increased (Figure 5j,n), and a clear elongation in fibroblast morphology is observed (Figure 5c,f), indicating a cellular reaction to the increase in substrate stiffness. After replacing the incubation solution with one containing 0% (w/v) PEG, the cell morphology gradually returns to the original round shape, with cells showing only slight elongation after three days without PEG, and complete recovery of the round cell morphology after seven days without PEG (Figure 5d,g). For more quantitative comparisons of these effects on cell morphology, refer to Section S7 (Supporting Information). Cell morphology was also affected by inverting this process (i.e., 10% to 0% to 10% 8 kDa PEG concentrations), with cells that initially had a stretched morphology becoming round after PEG removal, then recovering the stretched morphology (see Section S7, Supporting Information). This change and recovery of cell morphology by dynamic PEG concentration tuning demonstrates how this technique can be used to dynamically adjust polymer hydrogel mechanical properties (Figure 5i–k,m–o), which are then sensed by cells grown on them, influencing their morphology over time. This morphological change in cells with stiffness increases of 100–200 kPa (Figure 5j,n) is well in line with previous studies, where, depending on the cell type, stiffness changes as small as a dozen kPa can lead to dramatic morphological and/or behavioral changes, as is the case for cardiomyocytes,^[^ ^49^ ^]^ myoblasts,^[^ ^50^ ^]^ and even human pathogens such as *Acanthamoeba castellanii*.^[^ ^51^ ^]^ As such, this PEG interaction technique could provide a much‐needed means of dynamically tuning hydrogel stiffness during cell culture with minimal effort and cost.

To control for potential crowding effects on cells caused by dissolved PEG in the media, cells were grown directly on the plastic well without a hydrogel present and subjected to the exact same solution conditions as cells grown on alginate hydrogels (Figure 5h). Another control was performed by growing cells on the plastic well but with a hydrogel present in the well (Figure 5l). Morphologies for cells grown on the plastic well in the presence of PEG (Figure 5l) demonstrated less elongation than those grown on the hydrogel in the same well (Figure 5f). As these cells were exposed to the same medium, this indicates that PEG absorption and crowding on the cells are not responsible for cell morphology or behavioral differences. Instead, we assume that it is the PEG interaction‐induced change in hydrogel viscoelasticity that changes cell morphology. Furthermore, cells grown in wells with no alginate present and media containing 10% (w/v) 8 kDa PEG were permeabilized and died (Figure 5h). This supports the idea that when incubated in a PEG solution, hydrogels permit solvent to diffuse out of their matrices while simultaneously absorbing a large proportion of PEG molecules, thus protecting cells from any possible negative effects, such as crowding or osmotic pressure,^[^ ^52^ ^]^ caused by PEG exposure. RP‐HPLC analysis after 10 days of crowding supports this finding (Figure 3g), showing that ≈30–40% of the PEG remains in solution (Figure S24, Supporting information). Remarkably, under these conditions, the cells maintain viability at 30–40% of the 10% PEG in the cell culture medium, which is ≈2.5% of the 8 kDa PEG. This is consistent with our previous report showing that cell viability remains robust at levels above 90%.^[^ ^36^ ^]^

### 2.5 Stiffening Effects on the Cytoskeleton and Nucleus

**Figure 6:**
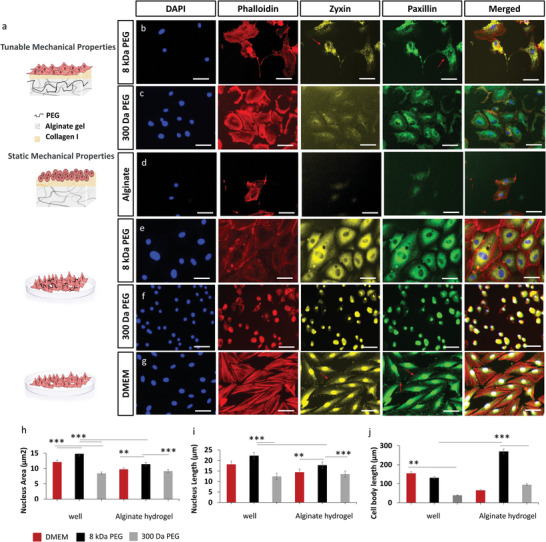
Cell attachment and morphology under dynamic PEG hydrogen bonding and different substrate conditions. a) Schematics: PEG interaction‐mediated control of cell attachment and spreading on the alginate hydrogels b) with 10% (w/v) 8 kDa PEG, and c) with 10% (w/v) 300 Da PEG; d) cells grown on alginate hydrogels without PEG, cells grown on a well plate treated with e) 10%(w/v) 8 kDa PEG‐DMEM and f) 10%(w/v) 300 Da PEG‐DMEM, where substrate stiffness is independent of PEG concentration, controlling for crowding effects on cell morphology and growth; g) positive control of untreated cells. Immunofluorescence staining with DAPI (blue, nucleus), Phalloidin (red, actin filaments), Zyxin (yellow, Focal adhesion), Paxillin (green, cytoskeletal protein that is integral to the formation of focal adhesion). Histograms represent h) average nucleus area (µm^2^), i) nucleus length (µm), j) cell body length (µm). Scale bars: 50 µm, number of independent experiments: 2, number of technical replicates per experiment: 3, number of images from which the shown representative images were chosen: 3. Data are presented as the mean ± standard deviation and analysed using the one‐way Analysis of Variance (ANOVA) with ^**^ *p* < 0.01 and ^***^ *p* < 0.001.

To visualize the stiffening effects on the cytoskeleton and nucleus, cells were grown either on alginate hydrogels or on a plastic well and incubated with or without PEG (300 Da or 8 kDa) in solution. They were then stained with one of several fluorescent markers (**Figure** **6**). Cells grown on alginate biofunctionalized with collagen type I in the presence of 8 kDa PEG demonstrate a more stretched morphology (Figure 6b), while those grown in 300 Da PEG (Figure 6c) or without PEG (Figure 6d) show a more rounded morphology, a clear difference between cells in both conditions. To verify that this stretched morphology is indeed due to increased stiffness, cells were grown on 7% (w/v) alginate, which had a similar *G*′ to PEG‐interacting 2% (w/v) alginate. Despite the absence of PEG present in the cell media or alginate hydrogel, the cells demonstrate similar morphologies to those grown on 2% (w/v) alginate that has interacted with PEG (Section S7, Supporting Information). Cells grown on plastic also show some slight differences in cell morphology between those exposed to PEG (Figure 6e,f), and those not exposed to it (Figure 6g). Cells incubated in a well with PEG but no hydrogel do not present any actin filaments, likely due to PEG penetration of the cells. While crowding due to PEG in solution will certainly affect cell morphology and behavior, the simple control of growing cells on plastic in the same well plate as the hydrogels allows crowding effects to be studied independently from stiffness changes. Crowding can also cause water diffusion out of cells, increasing cell stiffening and impacting stem cell differentiation,^[^ ^52^ ^]^ which may explain the differences in cell morphologies presented in Figure 6e–g.

Quantifications of nucleus area (Figure 6h), nucleus lengths (Figure 6i), and cell body length (Figure 6j) show interesting differences in cell morphology as a function of each condition, including differences between cells grown in the presence of both 300 Da and 8 kDa PEG on either alginate hydrogels or on plastic. Measurements of nucleus area show significant differences not only between cells grown on alginate either with or without PEG, cells grown directly on plastic with or without PEG, but also between both samples grown on either alginate or plastic in the same well. Nucleus area (Figure 6h) and length (Figure 6i) increase in the presence of 8 kDa PEG, but not in the presence of 300 Da PEG, perhaps as 300 Da PEG is more easily absorbed by cells, while the longer PEG is less so, allowing it to crowd cells more effectively, in turn affecting morphology. Cell body length, however, is strongly impacted by PEG‐induced stiffening (Figure 6j), independent of crowding/osmotic effects.

These cell results show that the PEG interaction technique applied to polymer hydrogels can clearly be sensed by cells grown on them, a critical cellular control factor when studying stem cell differentiation as it can be directed via changes in substrate stiffness.^[^ ^1^, ^4^ ^]^ It also can be done in a manner that minimally affects cell behavior and morphology.

## 3 Conclusion

Here, we demonstrate that PEG interacting with polymer hydrogels is a reversible, dynamic means of tuning cell culture scaffold stiffnesses. By changing the concentration of PEG in cell culture medium, the viscoelastic properties of the hydrogel can be controlled in situ, simply by changing the medium: increasing the PEG concentration increases material stiffness over a period of days. Reducing the PEG concentration in the medium or removing it all together decreases the material stiffness, removing PEG interactions with the alginate hydrogel polymers. This strategy of dynamically changing the concentration of PEG in solution to control hydrogel stiffness is a novel means of dynamically and reversibly changing the mechanical environment of cells by changing the cell culture medium. We posit that these PEG interactions are hydrogen bonds occurring between PEG and the hydrogels. Furthermore, it is applicable to several hydrogel types, has little effect on cell behavior when applied to hydrogels, and it is incredibly simple to apply. Due to the flexibility of this technique, it is of major interest to the biomedical and life sciences communities for future applications in mechanically controlled processes, such as organoid and tissue growth.

## 4 Experimental Section

### Materials

PBS (phosphate‐buffered saline) was purchased from Thermo Fisher Gmbh, Life Technologies. Polyethylene glycol (PEG) of all molecular weights and sodium alginate/alginic acid were purchased from Sigma–Aldrich.

### Alginate Hydrogel Preparation

Preparation of alginate hydrogels was performed by dissolving 1 g of sodium alginate/alginic acid in distilled water (50 mL) for a 2% (w/v) alginate solution. The reaction mixture was stirred and heated at 95 °C for 60 min. The volume of desired alginate was poured into a mold, petri dish, or well plates (depending on the final application). A pre‐wetted filter or glass Petri dish were placed on top of the alginate solution, depending on the final application, to ensure homogeneous crosslinking. For rheometry, a volume of 10 mL of 2% (w/v) alginate was used. To crosslink the alginate, a volume of 100 mm CaCl_2_, 25 mm HEPES solution at pH 7.5 was carefully pipetted on top of the filter or on top of the alginate solution under the glass Petri dish and left to crosslink overnight. After crosslinking, all hydrogels were kept in a 100 mm CaCl_2_, 25 mm HEPES solution at pH 7.5 to prevent dehydration and degradation. Until further usage, the samples were kept in the fridge at 4 °C.

Biofunctionalization of alginate hydrogels was accomplished by immersing 2% (w/v) alginate hydrogels in 100 mm 2‐(N‐morpholino)ethanesulfonic acid buffer (MES) for 20 min, then incubating the gels 80 min in 10 mM 1‐Ethyl‐3‐(3‐dimethylaminopropyl)carbodiimide (EDC) solution. The solution was then removed, with excess EDC removed by washing twice with 100 mm MES buffer for three min. per wash. Afterwards, the samples were washed two times with PBS buffer and one time with DMEM to achieve a pH level similar to the cellular environment. Until further use, the gels were kept in in filtered 100 mm CaCl_2_ with 25 mm HEPES, pH 7.5 solution at 4 °C. Next, hydrogel‐bonded EDC was attached to collagen type I by incubating the hydrogels in a 30 µg mL^−1^ collagen type I solution in PBS overnight at 4 °C. For further information regarding collagen functionalization, see the Cell Culture section below, and refer to Stanton, et al.^[^ ^53^ ^]^ and Grinnell and Ho.^[^ ^54^ ^]^ Until the cell‐seeding, alginate gels were immersed in a 100 mm CaCl_2_, 25 mm HEPES, pH 7.5 solution at 4 °C. Solutions of either 10% (w/v) 300 Da or 8 kDa PEG in DMEM with 25 mm HEPES, pH 7.5 were added to imitate experimental conditions performed previously, and kept at 4 °C.

### PEG‐Interaction Experiments

PEG interactions with polymer hydrogels were accomplished by immersing 10 mL hydrogels in 10 mL of PEG solution at the indicated molecular weight with 100 mm CaCl_2_, 25 mm HEPES solution at pH 7.5, and left in the fridge at 4 °C for the number of days indicated.

### Rheological Measurements

Rheometry measurements of the storage modulus (*G*′) and loss modulus (*G*″) for hydrogels were performed using a rotational rheometer equipped with an 8 mm parallel plate geometry (Kinexus pro+, NETZSCH Analyzing & Testing, DE). A cross‐linked hydrogel was placed on the rheometer for the measurements. An initial measuring of 1 N gap force was set and the moduli were measured as a function of frequency at 0.1% shear strain and controlled temperature of 37 °C (Peltier lower plate). To avoid drying of the sample during testing and to maintain the humidity, medium (in which the gels were kept) was added on top of the gels and deionized water was placed in the reservoir of the rheometer stamp in the Kinexus rheometer. For similar conditions in the Bohlin rheometer, a Petri dish was affixed to the bottom plate, the distance was zeroed, and, after laying down the gel, enough medium was added to reach nearly the height of the top plate such as not to interfere with rheometric measurements, as in Daalkhaijav & Walker.^[^ ^55^ ^]^ All rheological experiments were performed in six replicates. Refer to Figure S1 (Supporting information) for measurements of *G*′ and *G*″ versus frequency performed on a 2% (w/v) alginate hydrogel, as well as measurements of *G*′ at 1 Hz for several alginate hydrogel concentrations. For comparisons of 6% and 7% (w/v) alginate hydrogels with 2% (w/v) alginate hydrogels interacted with either 6 or 8 kDa 10% (w/v) PEG, refer to Figure S2 (Supporting information). For an explanation on how to convert rheometry data to Young's modulus, see Section S4 (Supporting Information), and for example data converted to Young's moduli refer to Figure 2c.

### Reversible PEG‐Interaction Experiments

Reversible PEG‐interaction experiments on alginate hydrogels were performed by leaving 2% (w/v) alginate hydrogels created without PEG present for 10 days, then measuring their viscoelastic properties on a rheometer. They were then placed in a 10% (w/v) 8 kDa PEG solution and left for an additional 10 days, where their viscoelastic properties were again measured on the same rheometer. This cycle was then repeated once. A similar cyclic experiment was then performed as before, except the alginate hydrogel was first incubated in 10% (w/v) 8 kDa PEG instead immediately after creation (Figure S4, Supporting information), and their viscoelastic properties measured after 10 days. Then the hydrogels were incubated in pure DMEM solution, and their viscoelastic properties were measured again on the same rheometer. This cycle was then repeated once.

### Microindentation Measurements

Microindentation measurements of PEG‐interacting alginate hydrogels were performed using a Pavone nanoindentation platform (Optics11 Life). Measurements required the adhesion of the gels to a flat surface, achieved by the preparation of a 5% agarose solution, which was heated to 100 °C using a microwave. Approximately 0.5 mL of this solution was poured into the wells of a 12‐well plate, and the prepared cylindrical hydrogels were placed in the center of each well, while the agarose solution was still hot. The well plates were then cooled to 4 °C for 20 min, to allow the agarose to gel and solution was added to the wells to keep the alginate/PEG‐alginate gels stable. A total of three samples of each hydrogel were prepared for each condition.

The measurements were carried out using a cantilever with 0.280 N m^−1^ stiffness and a 23.5 µm tip radius. Before the measurements were performed, the medium/PEG‐medium was removed and replaced by deionized water. After the device was calibrated, load control profiles were carried out with 1 µΝ load at a speed of 2 µm s^−1^ followed by a hold time of 0.5 s and subsequent retraction at 2 µm s^−1^ on each location. Data analysis was done using the Data Viewer (V2.5.0) software supplied by the device manufacturer. The Young's modulus *E* from each load‐indentation curve was calculated with the Hertzian contact model according to a publication of Huth et al.,^[^ ^56^ ^]^ using a constant indentation speed.^[^ ^57^ ^]^ The contact point of each load‐indentation curve was found by using the software integrated contact fit up to 50% of the maximum load. The Hertz fits were applied in the range between the contact point (0 µΝ) and 0.4 µΝ. Refer to Figure 2e,f for raw microindentation data, as well as the fit.

### Swelling Percentage Measurements

Swelling percentage measurements of hydrogels were performed by creating 2% (w/v) alginate hydrogels, as described above, then incubating them in water with or without 10% (w/v) 300 Da or 8 kDa PEG for 60 h at room temperature. Their swollen masses *m_s_* were then weighted (scale ≈100 mg), the hydrogels dried at 37 °C for 60 h, and their dry masses *m_d_* measured. Six replicates were performed for each condition, with the Swelling percentage calculated using $\%swelling=\left(m_{s}-m_{d}\right)\;/m_{d}\times 100\%$ ^[^ ^58^ ^]^

### Hydrolytic Stability Experiments

Hydrolytic stability of alginate hydrogels was performed on 2% (w/v) alginate hydrogels either with or without 10% (w/v) 300 Da or 8 kDa PEG set in Dulbecco's Modified Eagle Medium (DMEM), and stored at 4 °C. In the latter hydrogels, ample time was given to permit PEG to diffuse into the hydrogels. All hydrogels were kept in their respective media for 65 days at 37 °C in the incubator to mimic in vivo conditions. The degradation was analyzed by following the decay in the mass of the swollen gel as a function of the time.

### Analytical Reverse Phase High‐Performance Liquid Chromatography

Analytical reverse phase high‐performance liquid chromatography (RP‐HPLC) was performed with a Hitachi Primaide chromatography system containing a 1110 Pump, a 1210 auto sampler, 1310 column oven and a 1430 diode array detector. A VDS optilab VDSpher PUR 100 C18‐SE (250 mm × 4.6 mm, 100 Å, 5 µm) column and a flow rate of 1 mL min^−1^. On run is in general: 0 to 5 min isocratic (equilibration), 5 to 35 min gradient, 36 to 41 min isocratic (95% B in A to wash the column) and 42 to 50 min isocratic (equilibration).

### Confocal Microscopy

Confocal microscopy was performed with a Nikon A1R confocal microscope. Alginate hydrogels were created as per the protocol above with no PEG present, then their fluorescence was measured on a confocal microscope. PEG‐interacting alginate gels were prepared by incubating individual hydrogels in solutions containing 10% (w/v) 8 kDa fluorescent PEG (FITC‐PEG, from Abbexa Ltd), using 1 mL of FITC‐PEG solution per 50 mg gel. The gels were incubated for 10 days at 4 °C, to ensure an equilibrium concentration of FITC‐PEG molecules both inside and outside the alginate network before being measured on the confocal microscope. To demonstrate the FITC‐PEG diffusion inside the hydrogels (with/without FITC‐PEG); z‐stacks of hydrogels were imaged with the 483,7 laser (laser power: 30%, 20x objective, NA ≈617.6 nm, scan speed setting = 0.125, scan size = 1024).

### Cell Culture

Cell culture was performed using wild‐type mammalian rat embryonic fibroblasts (REF‐52 wt; B. Geiger, Weizmann Institute, Israel), and were cultured in RPMI 1640 medium (PAN Biotech, P04‐04515) supplemented with 10% Fetal Bovine Serum (PAN Biotech, P30‐3032) and 1% antibiotics (penicillin/streptomycin) (Invitrogen), at 37 °C under a humidified atmosphere containing 5% CO_2_. Cell culture media was changed every second day. Cells from passage 8–15 were used.

Hydrogels on which cells were grown were cut out with a biopsy punch (diameter 8 mm), then placed into 48 well plates. Hydrogels were sterilized by immersing them in 70% ethanol and leaving them for at least 15 min. Ethanol was removed by incubating the hydrogels for 5 min in sterile PBS while shaking, three times. The hydrogels were then exposed for 30 min to UV light for extra sterilization, then stored in a fridge overnight at 4 °C in filtered 100 mm CaCl_2_ with 25 mm HEPES (pH 7.4). A 2‐(N‐morpholino)ethanesulfonic acid (MES) solution was prepared by weighing out 0.39 g MES into a falcon tube, dissolved in 20 mL DIH_2_O for a total concentration of 19.5 mg mL^−1^, then sterilized by syringe‐filtering it into a new 50 mL falcon tube. This MES buffer was used to rinse the hydrogel samples before the crosslinking reaction.

For the collagen‐hydrogel reaction, 40 mg of EDC‐HCl was weighed into a falcon tube, dissolved in 20 mL DIH_2_O for a concentration of ≈0.1 m EDC, and sterilized by syringe‐filtering into a new 50 mL falcon tube. Sterile MES buffer was added to each hydrogel sample, ensuring that they were completely covered, then left to incubate for at least 15 min. The MES buffer was removed from the hydrogel samples, and replaced with 5 mL of 0.1 m EDC, enough to completely cover them. EDC solution was left to interact with the hydrogels for at least 1 h in the 4 °C fridge. After this, the EDC solution was removed from the hydrogels and rinsed with MES buffer by shaking them for 2 min, 3 times to remove any remaining unreacted EDC. Hydrogels were washed 3 times with PBS, then left in filtered 100 mm CaCl_2_ with 25 mm HEPES (pH 7.4) in the 4 °C fridge.

Hydrogels were coated in collagen type I (30 µg mL^−1^ in PBS with 1 µL mL^−1^ acetic acid) overnight (later ± PEG). Hydrogels were washed with sterile PBS for 3 × 5 min to remove any excess protein, then washed 3 × 5 min with DMEM. Finally, hydrogels were left in 100 mm CaCl_2_ with 25 mm HEPES (pH 7.4) at 37 °C.

REF52wt cells were taken from their current passage and counted. Collagen‐coated hydrogels were seeded with REF52wt cells by pipetting ≈20 000 cells onto their top surface. DMEM with 10% FBS was added to the hydrogel samples with no PEG present, and then placed in an incubator at 37 °C.

PEG of all molecular weights was sterilized in ethanol, dried, and then dissolved in DMEM cell medium with 10% FBS + 1% P/S with 10% PEG (PEG‐DMEM). HEPES was dissolved in the solution to obtain a pH between 7.2 and 7.5. Hydrogels with seeded cells were exposed to their respective experimental medium, depending on whether the samples were to be exposed to PEG‐DMEM or DMEM without PEG, then left to incubate at 37 °C. Every 2 days, cells were imaged in the fluorescence microscope. Every 2 days, the media were exchanged with either PEG‐DMEM or DMEM with no PEG, as a function of whether the hydrogels and cells were to be exposed to PEG. Cells were fixed using a 5 h wash of 4% PFA with PBS 3x times, then left in a 4 °C fridge.

### Live/Dead Assays

Live/dead assays based on calcein AM (Sigma, C1359‐100UL) and propidium iodide (ThermoFisher, P3566) were carried out following the manufacturer's protocol, where live cells are stained green (excitation/emission ≈490/515 nm) and dead cells appear red (excitation/emission ≈535/617 nm). For control experiments, untreated cells (cells incubated in the absence of hydrogel) were used as positive controls (viable cells), and cells treated with ethanol for 5 min were used as negative controls (dead cells). The cells were imaged with an Olympus IX81 inverted microscope using an Olympus UCPlanFLN 20x/0.70 or CAchN 10x/0.25 PhP Objectives and a Hamamatsu Orca‐2 camera and counted using ImageJ software. Cell viability was calculated by % *Cell* *Viability*  =  (*total* *n*° *of* *cells*  −  *n*° *of* *stained* *red* *cells*)/(*total* *n*° *of* *cells*)*x* 100%.^[^ ^59^ ^]^ Example images of controls are provided in Figure S3 (Supporting information).

### Dynamic Hydrogel Tuning During Cell Culture

Dynamic hydrogel tuning during cell culture was performed using 2% (w/v) alginate hydrogels functionalized with 30 µg mL^−1^ Collagen type I overnight. Fibroblast cells (20 000 per well (6 mm)) were seeded on top of the gels and incubated in 10% (w/v) 8 kDa PEG‐DMEM solution (25 mm HEPES, pH 7.5) for 10 days (Figure S4, Supporting information). Then, the PEG‐ DMEM solution was replaced with cell culture medium (25 mm HEPES, pH 7.5) and left for 10 days. This cycle was then repeated once more.

### Immunofluorescent Staining

Immunofluorescent staining was performed by leaving cells for 16 h in a 3% paraformaldehyde (PFA, from Sigma–Aldrich) solution to crosslink the cell membrane and cytoplasmic proteins, stabilizing cell morphology and allowing the used antigens access to the cells. The PFA solution was subsequently removed, and the gels were washed with PBS three times for five min. To make the cells more permeable, samples were treated with 0.2% PBS‐triton X‐100 for 10 min and washed with PBS three times for five min. To reduce background fluorescence in the samples, samples were incubated for 1 h at room temperature (RT) on a shaker in a blocking solution consisting of 0.1% PBS‐tween, 10% FBS, and 3% BSA, after which the blocker was removed. The primary antibody staining solution was prepared in blocking solution with a 1:250 dilution of Paxillin (cytoskeletal protein that is integral to the formation of focal adhesion; Catalog # ΑΗΟ0492, Invitrogen) and 1:100 dilution of Zyxin (focal adhesion; Catalog # Z4751, Merck), which was then added to the samples. Samples were kept overnight at 4 °C and were covered with aluminum foil to prevent photobleaching. The next day, the medium was removed, and the wells were washed with 0.1% PBS‐tween three times for five min. Using paxillin from mouse tissue and zyxin from rabbit tissue, the primary antibodies attached to the proteins. In the next step, fluorophore‐connected secondary antibodies must bind to their corresponding primary antibodies: secondary anti‐mouse antibody binds specifically to the primary paxillin antibody that comes from mouse tissue, and a similar process occurs with anti‐rabbit and zyxin that comes from rabbit. For the secondary antibody staining solution, the rhodamine phalloidin (actin filaments; Catalog # ab235138, Abcam) stain, and antibodies for both anti‐mouse and anti‐rabbit were diluted 1:1000 in blocking solution, added to the samples, and were kept covered in aluminum foil for 1 h at RT on the shaker. Medium was removed and the samples were washed with 0.1% PBS tween three times for five min. Finally, the DNA staining solution with Hoechst was prepared in a 1:1500 dilution in 0.1% PBS tween and added to the samples for 20 min. Again, gels and positive control were washed with PBS three times for five min and kept covered with aluminum foil in the fridge at 4 °C until use.

### Statistical Analysis

Statistical analysis was performed using Excel Data Analysis or Origin software. All the results are reported as the mean ± standard deviation. Rheology measurements used 5 hydrogels, while cell experiment statistics involved 3 independent experiments involving 3 replicates. Statistical differences were analyzed based on the one‐way Analysis of Variance (ANOVA). ^*^ *p* < 0.05, ^**^ *p* < 0.01, and ^***^ *p* < 0.001 values were used for significance.

## Conflict of Interest

The authors declare no conflict of interest.

## Author Contributions

S.S. and M.V. are co‐first/equal authors. F.C., A.D.l.C.G., L.T., L.T., and K.S. are co‐author. S.S. and M.V. contributed equally to this work. C.S. conceived and supervised the project, and acquired funding. S.S. conceived and designed the project. S.S. contributed in the materials development and rheological measurements. M.V. designed the project, performed most of the experimental work, and supervised L.T., L.T. and A.C.G. F.C. designed and performed the microindentation measurements. L.T. and L.T. performed the material preparation, rheological measurements and immunofluorescent stanning. S.S., M.V., F.C., and A.C.G. participated in data analysis. The manuscript was written through the contribution of S.S., M.V. and C.S. K.S. developed the collagen‐alginate biofunctionalization technique used. All authors have given approval to the final version of the manuscript. C.S. and M.V. were supported through the Max Planck School Matter to Life supported by the German Federal Ministry of Education and Research (BMBF), Max Planck Society and the Flagship Initiative “Engineering Molecular Systems”. We also acknowledge funding by the DFG under Germany's Excellence Strategy 2082/1‐390761711 (3D Matter Made to Order) and the Carl Zeiss Foundation, as well as the European Research Council through the Consolidator Grant PHOTOMECH (no. 101001797) and the Volkswagen Foundation through the Initiative “Life?,” Az. 96733. K.S. and C.S. thank the German Research Foundation for funding through the RTG 2154. C.S. and A.G. thank the DFG, SFB 1261 Biomagnetic Sensing (Project B7) for financial support. S.S. acknowledges Kiel Nano, Surface and Interface Science (KiNSIS).

## Supporting information



Supporting Information

## Data Availability

The data that support the findings of this study are available from the corresponding author upon reasonable request.
