# Supplementary material for: Dynamic and Reversible Tuning of Hydrogel Viscoelasticity by Transient Polymer Interactions for Controlling Cell Adhesion
Source: Adv Mater. 2025 Feb 11;37(12):2408616. doi: 10.1002/adma.202408616 (PMC11938001; doi:10.1002/adma.202408616)
Supplement: Supplementary file 1 — Supporting Information [file ADMA-37-2408616-s001.pdf]

ADVANCED MATERIALS

Supporting Information

for *Adv. Mater.*, DOI 10.1002/adma.202408616

Dynamic and Reversible Tuning of Hydrogel Viscoelasticity by Transient Polymer Interactions for Controlling Cell Adhesion

*Shane Scott, Maria Villiou, Federico Colombo, Angeles De la Cruz-García, Leon Tydecks, Lotta Toelke, Katharina Siemsen and Christine Selhuber-Unkel**

Supporting Information

Dynamic and Reversible Tuning of Hydrogel Viscoelasticity by Transient Polymer Interactions for Controlling Cell Adhesion

Shane Scott[×]*, Maria Villiou*[×]*, Federico Colombo, Angeles De la Cruz Garcia, Leon Tydecks, Lotta Toelke, Katharina Siemsen, Christine Selhuber-Unkel**

Dr. S. Scott

Department of Materials Science and Engineering, McMaster University, 1280 Main St. W., L8S 4L8, Hamilton, Ontario, Canada

Dr. M. Villiou, Dr. F. Colombo, A. De la Cruz Garcia, L. Tydecks, L. Toelke, Prof. C. Selhuber-Unkel

Institute for Molecular Systems Engineering and Advanced Materials (IMSEAM), Heidelberg University, D-69120 Heidelberg, Germany

E-mail: Selhuber@uni-heidelberg.de

Dr. M. Villiou, Prof. C. Selhuber-Unkel

Max Planck School Matter to Life, Heidelberg University, Jahnstraße 29, 69120 Heidelberg, Germany

Dr. M. Villiou

Max Planck Institute for Polymer Research, Ackermannweg 10A, 55128 Mainz, Germany

Dr. K. Siemsen

Institute for Materials Science, Kiel University, Kaiserstraße 2, 24143 Kiel, Germany

[×]These authors contributed equally to this work

*Corresponding author

Table of contents

Section	Page
1. Rheological Characterization of Alginate Hydrogels: Shear Moduli	3
2. Rheological Comparison of Different PEG Concentrations	18
3. Rheological Comparison of Different Alginate Concentrations vs PEG Interacting Hydrogels	19
4. Solvent Effects on Hydrogels	19
5. Rheological Characterization of PEG-interacting and Standard Agarose Hydrogels	21
6. Live/Dead Cell Assays	23
7. Dynamic Tuning of Cell Adhesion and Expansion on the Alginate Hydrogels	23

8. Cells Grown on High Stiffness Alginate Hydrogels with No PEG vs Those Grown in the Presence of PEG	28
9. HPLC Calibration Curve	30
10. Cells Grown on Fibronectin-functionalized vs Collagen-functionalized Alginate	32
References	32

1. Rheological Characterization of Alginate Hydrogels

Rheometry measurements using an 8 mm parallel plate geometry (Kinexus pro+, NETZSCH Analyzing & Testing, DE) were performed to measure the storage modulus (G') and loss modulus (G'') vs frequency in Hz, as in **Fig. S1a**. Hydrogel samples were placed between the plates, with 2 drops of deionized water put on top each to prevent drying of the samples. As the water on the surface reduced friction between the hydrogels and rheometer plates, slip occurred between the two producing a spike in both G' and G''. Measurements of these values at 1 Hz are used in the main text, and both this data and the artifact caused by slip can be seen in **Fig. S1b**. Testing of alginate hydrogels as a function of alginate concentration were performed, with the data at 1 Hz shown in **Fig. S1c**.

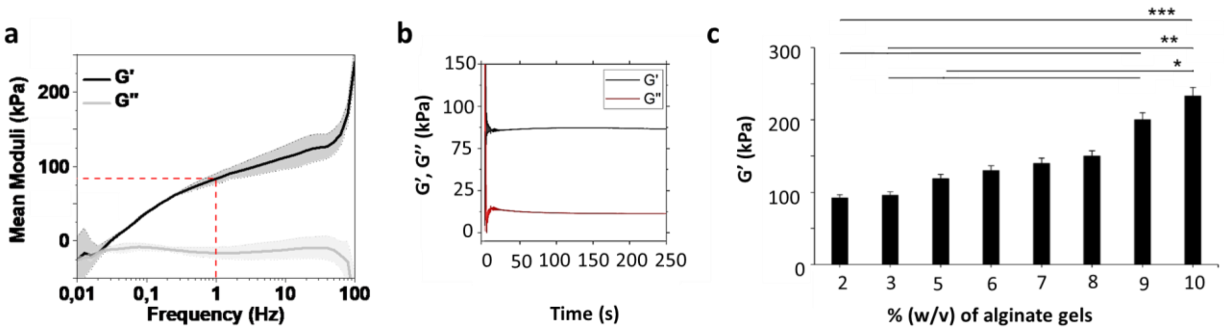

Figure S1. Rheological characterization of $CaCl_2$ cross-linked alginate gels (**(a)** 2% (w/v) alginate gel as measured by oscillatory rheology at constant pH 7.5, frequency sweep 0.01-100 Hz, 0.1% shear strain and 37°C; **(b)** 2% (w/v) alginate gel, **(c)** different alginate concentration), as measured by oscillatory rheology at constant pH 7.5, 1 Hz frequency, 0.1% shear strain and 37°C. Number of gels for each condition: 6

Strain sweep measurements were also performed to determine the ideal strain to use for 0.1%-7% alginate hydrogels incubated in 10% (w/v) 8 kDa PEG, and subsequent results after "washing" (i.e., incubating in cell media without PEG), allowing for accurate comparison between these

conditions:

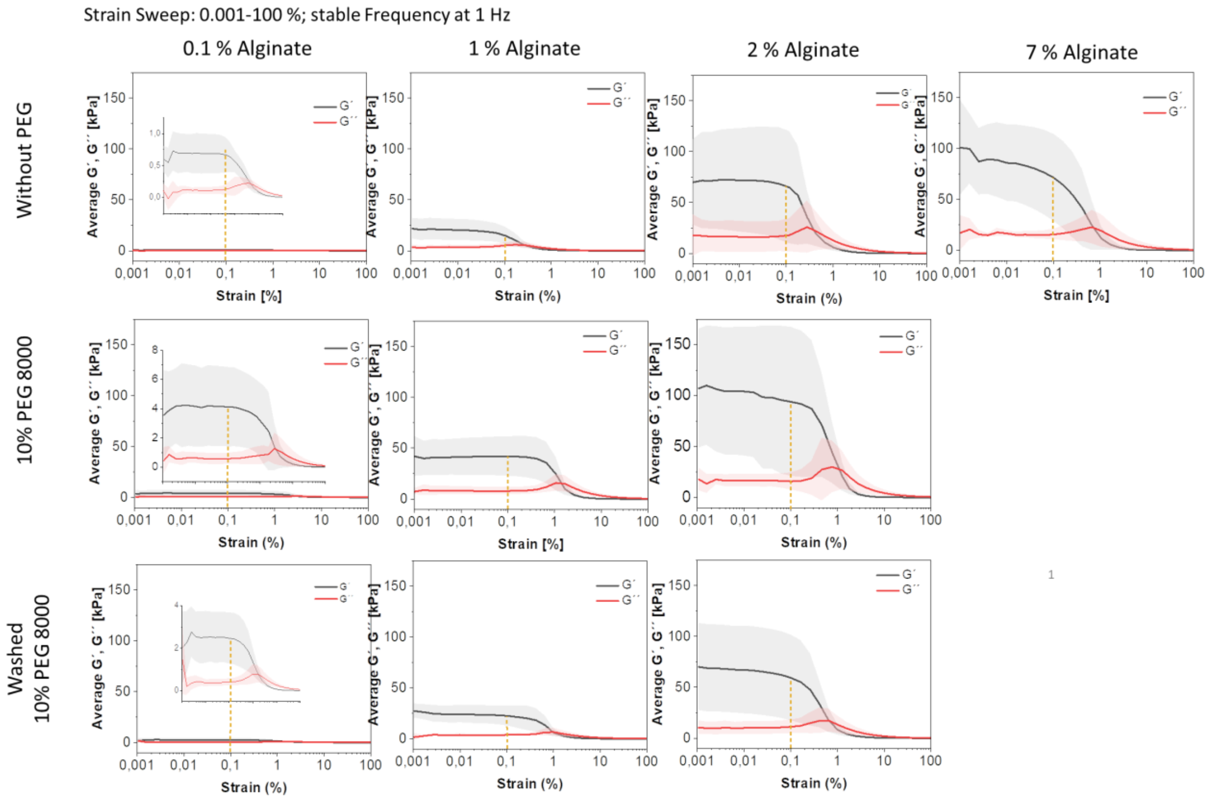

Figure S2. Rheological strain sweeps of $CaCl_2$ cross-linked alginate gels incubated with and without 10% (w/v) 8 kDa PEG. Alginate hydrogels with and without PEG incubation are measured by strain-sweep rheology at constant pH 7.5, 1 Hz frequency, and 37°C. Washed alginate hydrogels are those that were previously incubated in 10% (w/v) 8 kDa PEG, then incubated in cell media without the presence of PEG. Number of gels per condition: 5.

Rheometry data presented in the main paper are the result of frequency sweep measurements performed for 0.1%-7% alginate hydrogels incubated in 10% (w/v) 8 kDa PEG, and subsequent results after "washing" (i.e., incubating in cell media without PEG):

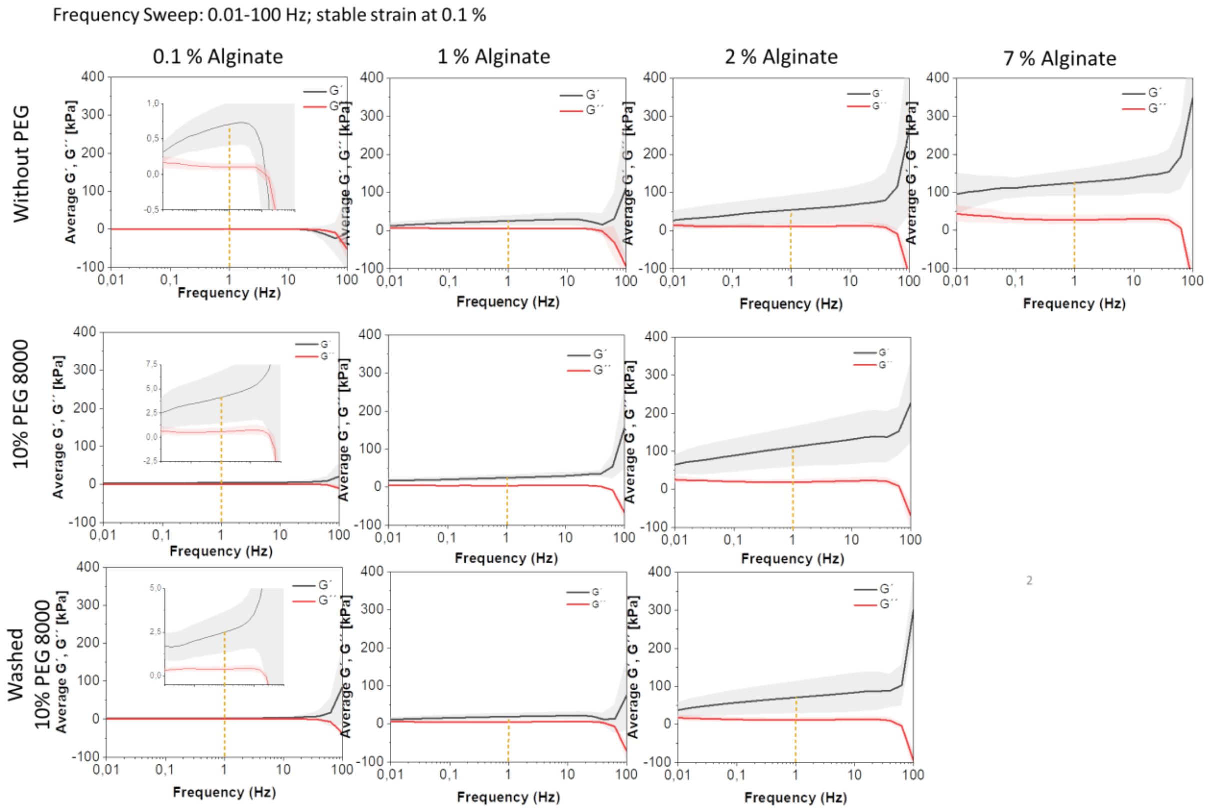

Figure S3. Rheological frequency sweeps of $CaCl_2$ cross-linked alginate gels incubated with and without 10% (w/v) 8 kDa PEG. Alginate hydrogels with and without PEG incubation are measured by frequency-sweep rheology at constant pH 7.5, 0.1% strain, and 37°C. Washed alginate hydrogels are those that were previously incubated in 10% (w/v) 8 kDa PEG, then incubated in cell media without the presence of PEG. Number of gels per condition: 5.

Frequency sweep measurements were also performed for 2% alginate hydrogels incubated in 10% (w/v) PEG at several molecular weights (0 kDa PEG, 300 kDa PEG, 600 kDa PEG, 1,500 kDa PEG, 3,000 kDa PEG, 6,000 kDa PEG, 8,000 kDa PEG, and 35,000 kDa PEG):

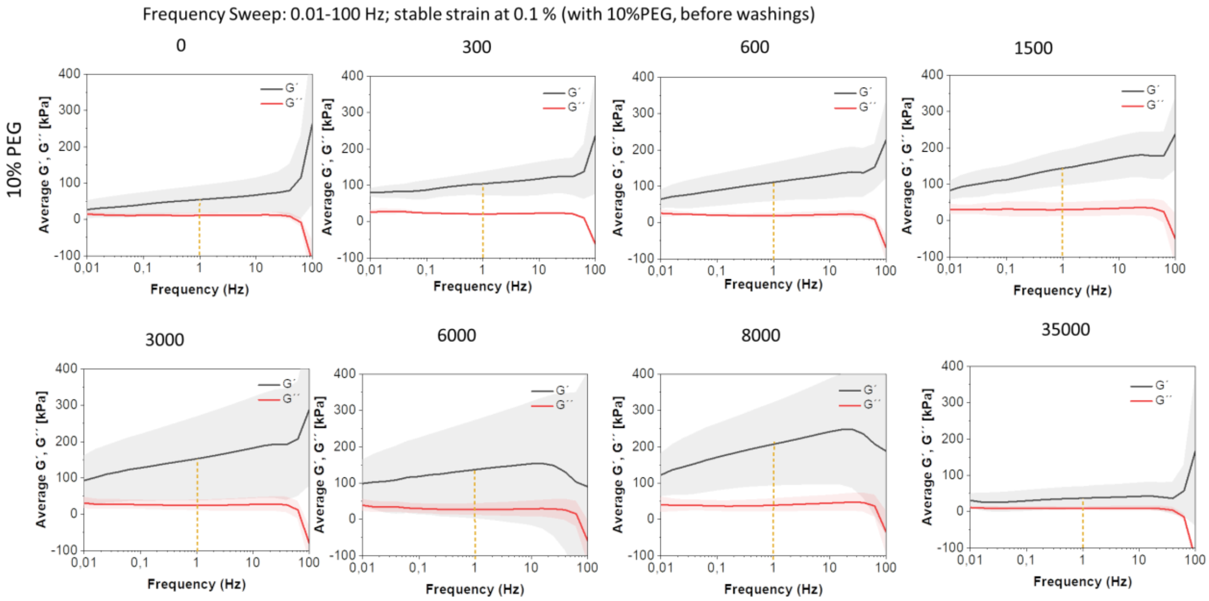

Figure S4. Rheological frequency sweeps of $CaCl_2$ cross-linked alginate gels incubated 10% (w/v) PEG at a range of PEG molecular weights (MW): 0 kDa, 300 kDa, 600 kDa, 1,500 kDa, 3,000 kDa, 6,000 kDa, 8,000 kDa, and 35,000 kDa. Alginate hydrogels with PEG incubation are measured by frequency-sweep rheology at constant pH 7.5, 0.1% strain, and 37°C. Number of gels per condition: 5.

After incubation in 10% PEG, the 2% alginate hydrogels were subsequently incubated in cell media with no PEG, and frequency sweeps were performed using a rheometer:

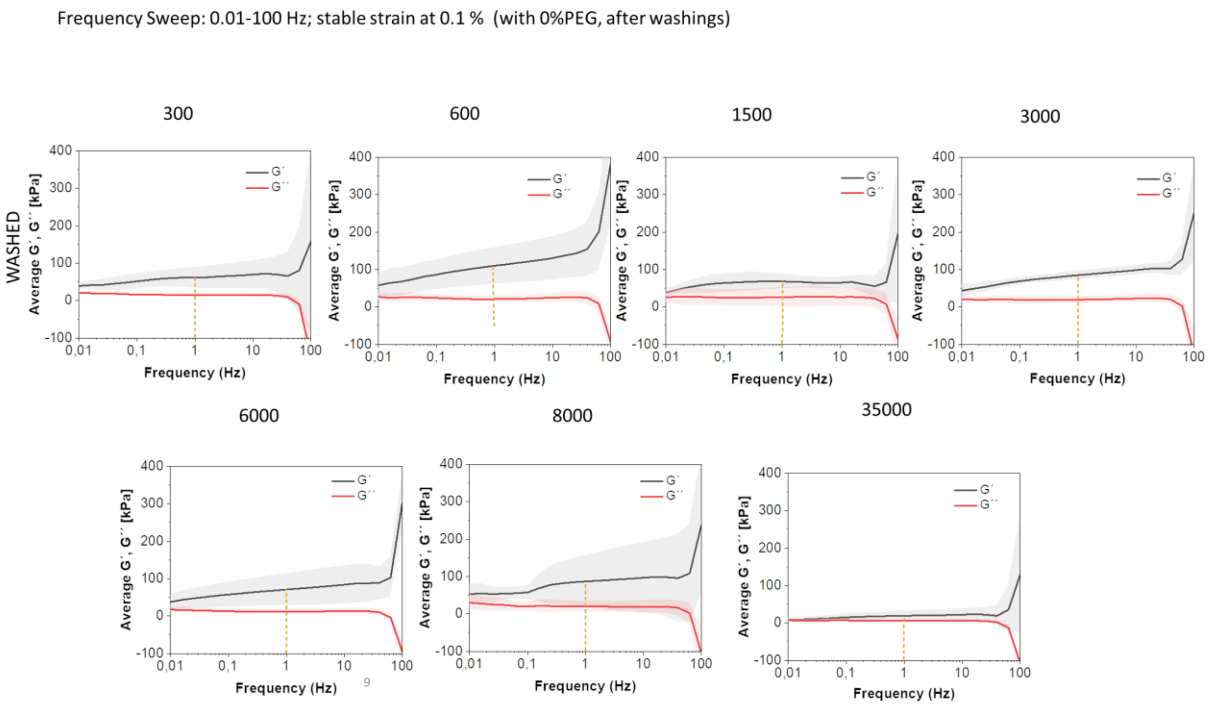

Figure S5. Rheological frequency sweeps of $CaCl_2$ cross-linked alginate gels incubated in cell media with no PEG after previous incubation in 10% (w/v) PEG at a range of PEG molecular weights (MW): 0 kDa, 300 kDa, 600 kDa, 1,500 kDa, 3,000 kDa, 6,000 kDa, 8,000 kDa, and 35,000 kDa. Washed alginate hydrogels are measured by frequency-sweep rheology at constant pH 7.5, 0.1% strain, and 37°C. Number of gels per condition: 5.

For comparison, the data of 2% alginate hydrogels incubated in 10% (w/v) PEG at a range of PEG MW in Fig. S4 and the same hydrogels after incubation in no PEG, as shown in Fig. S5, are presented in a single graph.

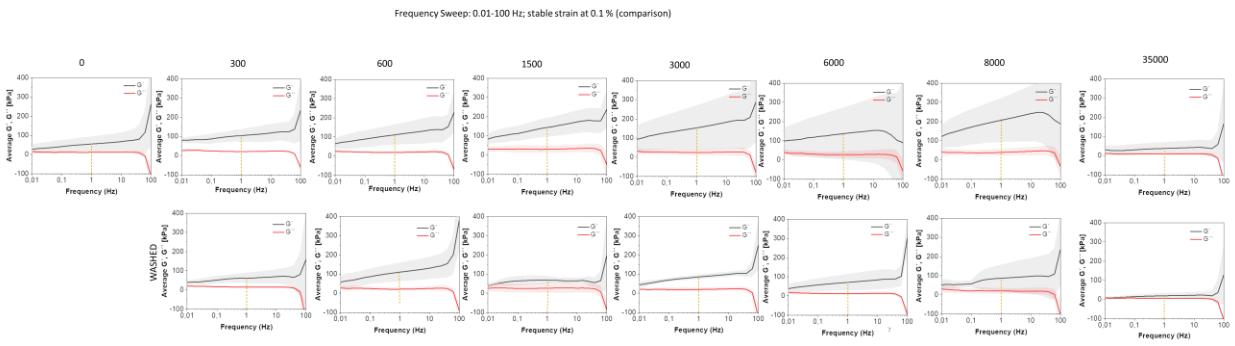

Figure S6. Rheological frequency sweeps of $CaCl_2$ cross-linked alginate gels incubated in (*above)* 10% (w/v) PEG at a range of PEG molecular weights (MW) (0 kDa, 300 kDa, 600 kDa, 1,500 kDa, 3,000 kDa, 6,000 kDa, 8,000 kDa, and 35,000 kDa) in comparison to these same alginate hydrogels (*below)* after incubation in cell media with no PEG. Washed alginate hydrogels are measured by frequency-sweep rheology at constant pH 7.5, 0.1% strain, and 37°C. Number of gels per condition: 5.

Strain sweeps of these same hydrogels incubated in 10% (w/v) PEG at a range of PEG MW (0 kDa, 300 kDa, 600 kDa, 1,500 kDa, 3,000 kDa, 6,000 kDa, 8,000 kDa, and 35,000 kDa) are also shown below.

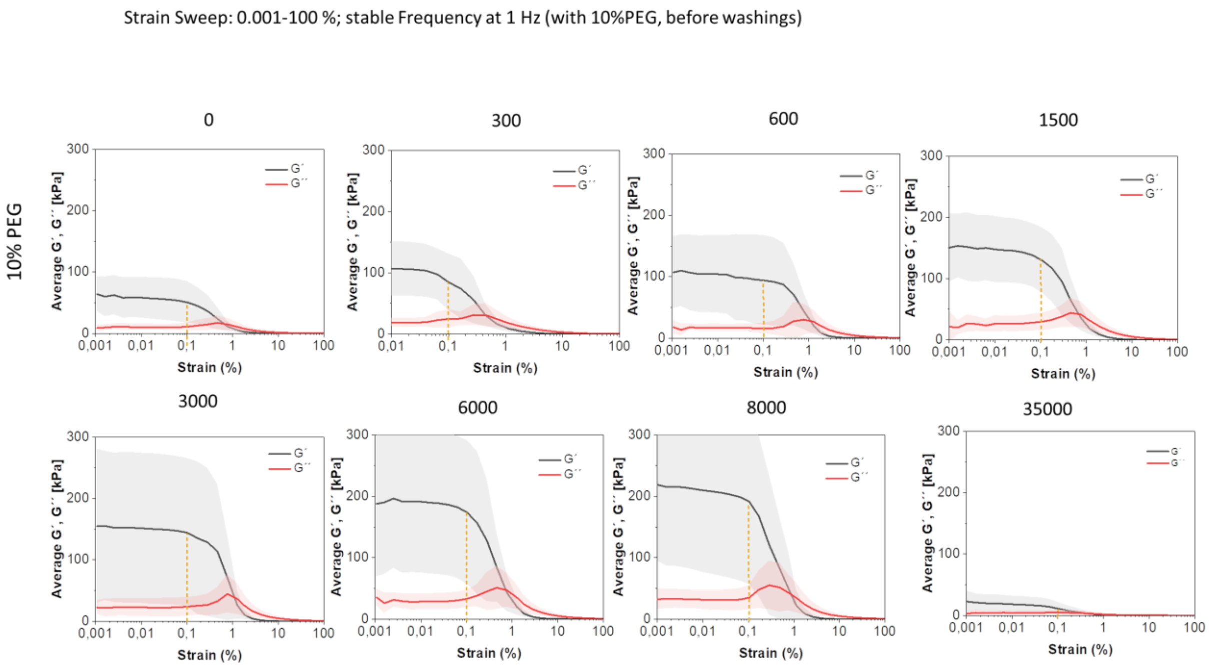

Figure S7. Rheological strain sweeps of $CaCl_2$ cross-linked alginate gels incubated with 10% (w/v) PEG at a range of PEG molecular weights (MW) (0 kDa, 300 kDa, 600 kDa, 1,500 kDa, 3,000 kDa, 6,000 kDa, 8,000 kDa, and 35,000 kDa). Alginate hydrogels incubated with PEG are measured by strain-sweep rheology at constant pH 7.5, 1 Hz frequency, and 37°C. Number of gels per condition: 5.

Strain sweeps of these same hydrogels incubated cell media with no PEG after previous incubation in 10% (w/v) PEG at a range of PEG MW (0 kDa, 300 kDa, 600 kDa, 1,500 kDa, 3,000 kDa, 6,000 kDa, 8,000 kDa, and 35,000 kDa) are shown.

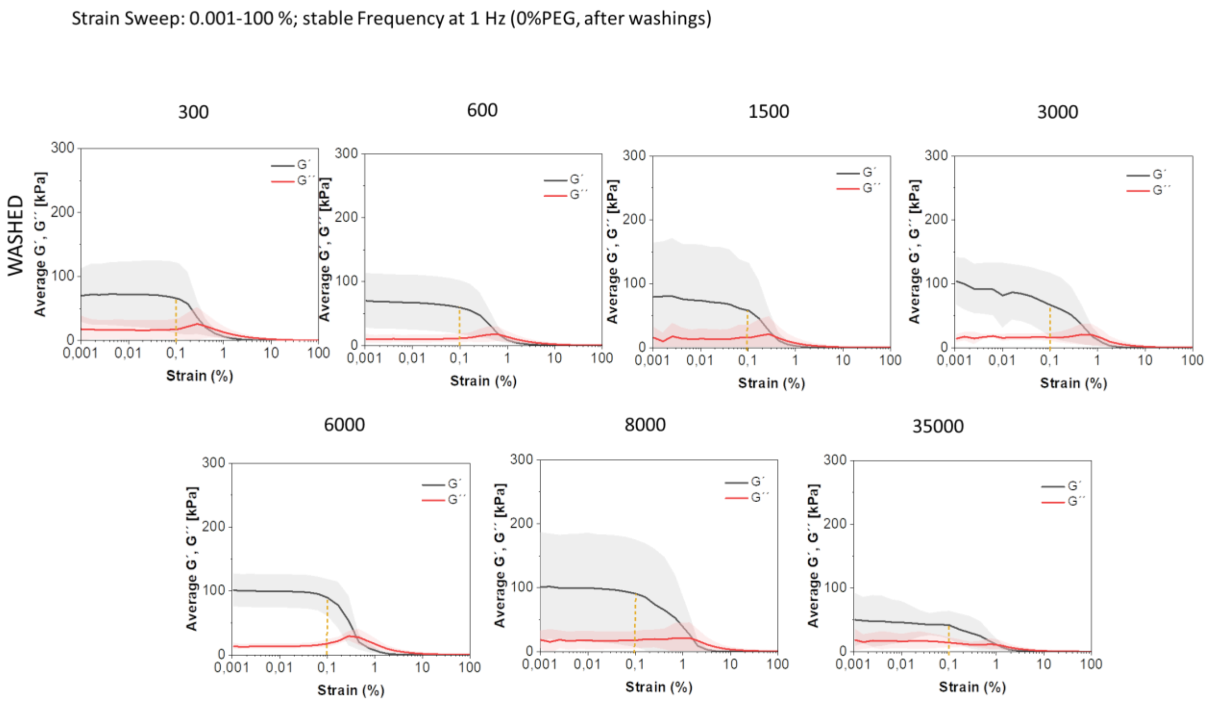

Figure S8. Rheological strain sweeps of $CaCl_2$ cross-linked alginate gels incubated with 10% (w/v) PEG at a range of PEG molecular weights (MW) (0 kDa, 300 kDa, 600 kDa, 1,500 kDa, 3,000 kDa, 6,000 kDa, 8,000 kDa, and 35,000 kDa). Alginate hydrogels incubated with PEG are measured by strain-sweep rheology at constant pH 7.5, 1 Hz frequency, and 37°C. Number of gels per condition: 5.

For comparison, the data of 2% alginate hydrogels incubated in 10% (w/v) PEG at a range of PEG MW in Fig. S7 and the same hydrogels after incubation in no PEG, as shown in Fig. S8, are presented in a single graph.

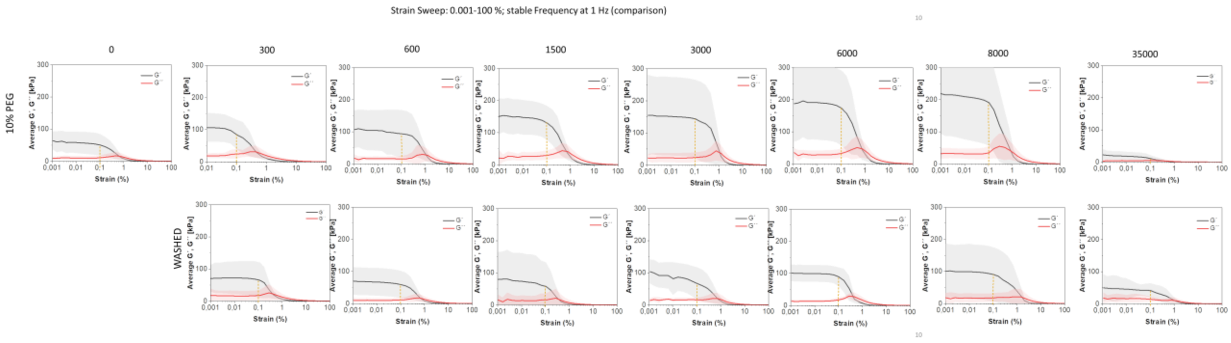

Figure S9. Rheological strain sweeps of $CaCl_2$ cross-linked alginate gels incubated in (*above)* 10% (w/v) PEG at a range of PEG molecular weights (MW) (0 kDa, 300 kDa, 600 kDa, 1,500 kDa, 3,000 kDa, 6,000 kDa, 8,000 kDa, and 35,000 kDa) in comparison to these same alginate hydrogels (*below)* after incubation in cell media with no PEG. Washed alginate hydrogels are measured by strain-sweep rheology at constant pH 7.5, 1 Hz frequency, and 37°C. Number of gels per condition: 5.

Stress relaxation measurements were carried out to investigate how quickly alginate hydrogels, with concentrations ranging from 0.1% to 7%, relax or return to their original state after being subjected to deformation. These hydrogels were first treated with a 10% (w/v) solution of 8 kDa polyethylene glycol (PEG). Further measurements were then taken after a 'wash' step in which the hydrogels were exposed to cell media without PEG.

The purpose of this experimental design was to understand the relaxation time of the hydrogels under the influence of PEG and to observe how the relaxation behaviour changed when the PEG was removed. This "washing" step involved exposing the hydrogels to a medium without PEG, essentially simulating conditions without the external influence of the PEG substance.

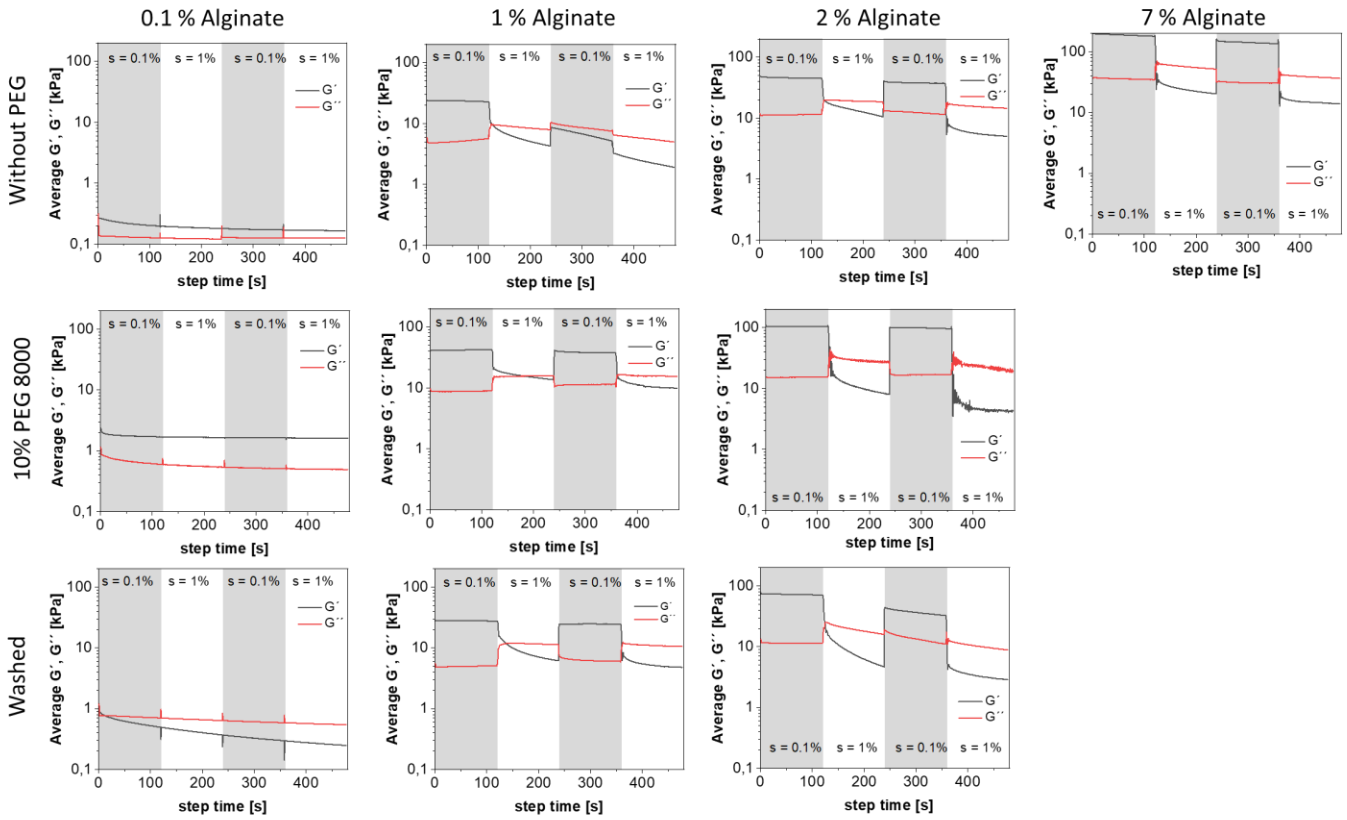

Figure S10. Rheological stress relaxation experiments of $CaCl_2$ cross-linked alginate gels incubated with and without 10% (w/v) 8 kDa PEG. Alginate hydrogels with and without PEG incubation are measured at constant pH 7.5, 0.1% to 1% strain, 1 Hz frequency and 37°C. Washed alginate hydrogels are those that were previously incubated in 10% (w/v) 8 kDa PEG, then incubated in cell media without the presence of PEG. Number of gels per condition: 5.

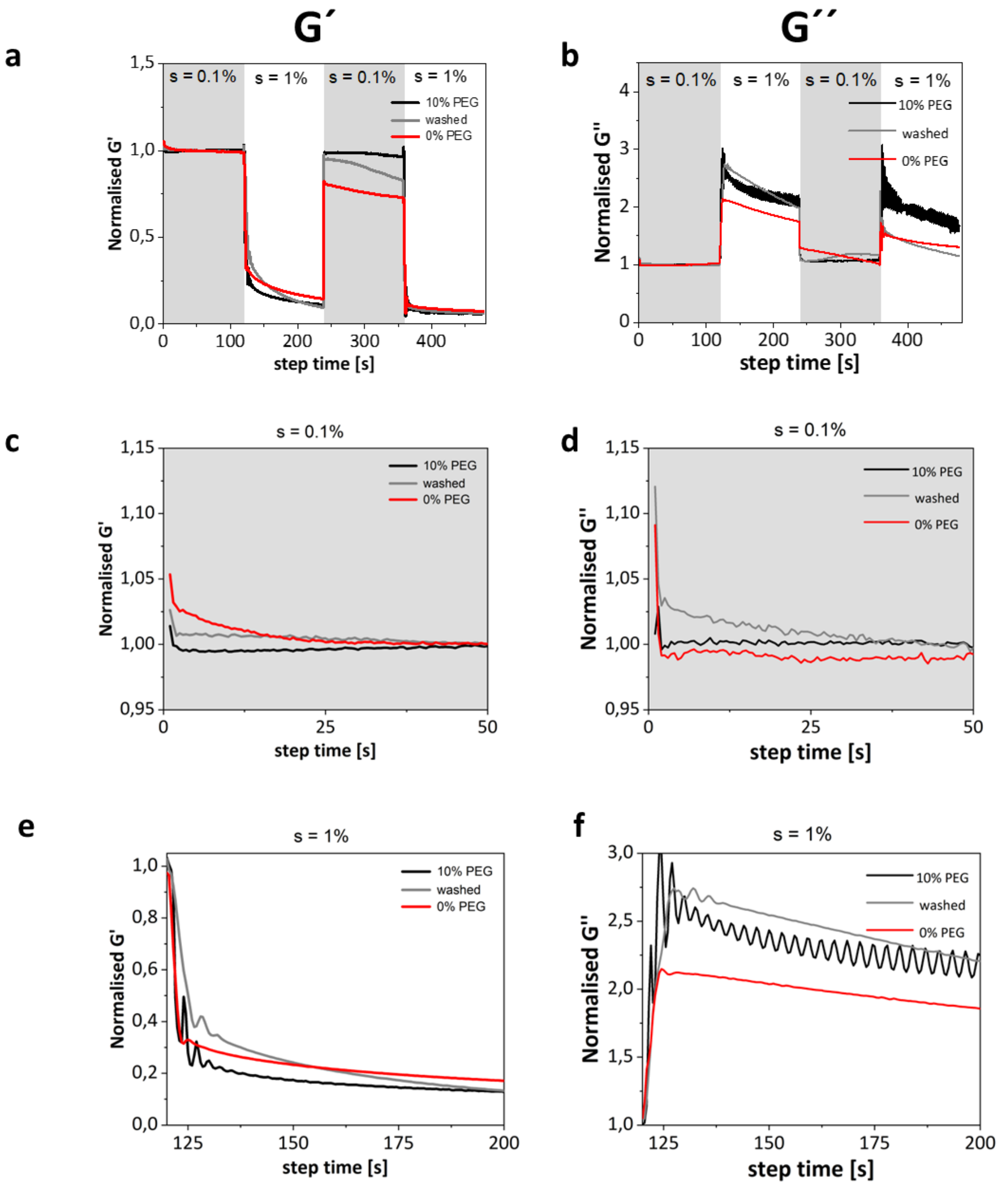

Figure S11. Rheological stress relaxation experiments of $CaCl_2$ cross-linked alginate gels incubated with and without 10% (w/v) 8 kDa PEG. Alginate hydrogels with and without PEG incubation are measured at constant pH 7.5, 0.1% to 1% strain, 1 Hz frequency and 37°C. Washed

alginate hydrogels are those that were previously incubated in 10% (w/v) 8 kDa PEG, then incubated in cell media without the presence of PEG. **(c-f)** Normalised G’, G’’ at strain 0,1% and 1%. Number of gels per condition: 5.

Stress relaxation measurements were also performed for 2% alginate hydrogels incubated in 10% (w/v) PEG at several molecular weights (0 kDa PEG, 300 kDa PEG, 600 kDa PEG, 1,500 kDa PEG, 3,000 kDa PEG, 6,000 kDa PEG, 8,000 kDa PEG, and 35,000 kDa PEG):

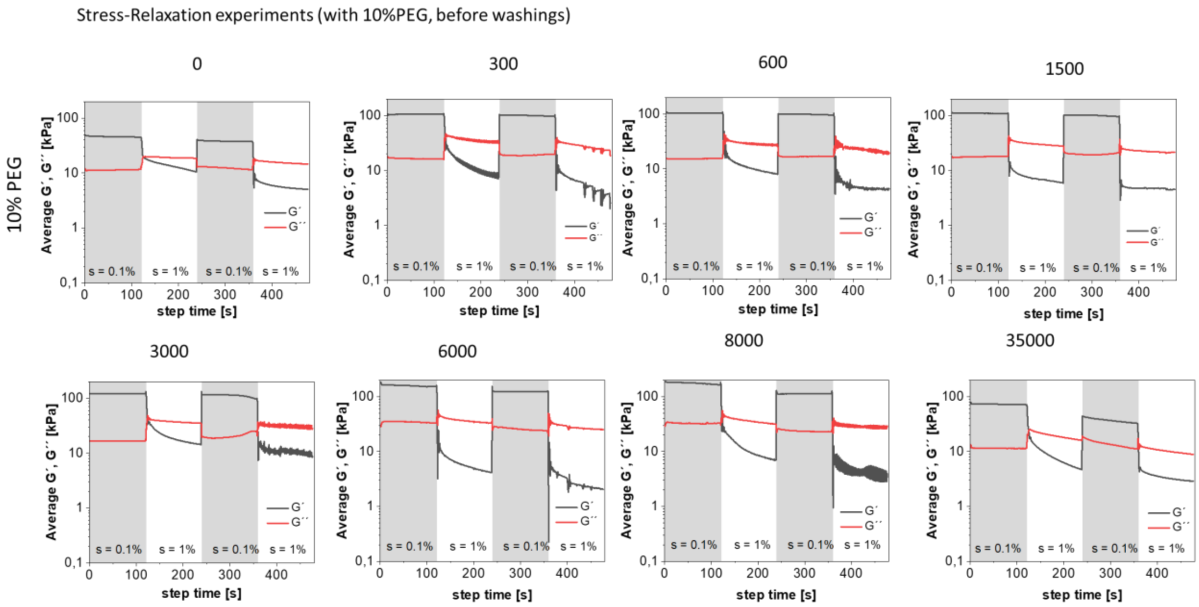

Figure S12. Rheological stress relaxation experiments of $CaCl_2$ cross-linked alginate gels incubated with 10% (w/v) PEG at a range of PEG molecular weights (MW) (0 kDa, 300 kDa, 600 kDa, 1,500 kDa, 3,000 kDa, 6,000 kDa, 8,000 kDa, and 35,000 kDa). Alginate hydrogels incubated with PEG are measured at constant pH 7.5, 0.1% to 1% strain, 1 Hz frequency, and 37°C. Number of gels per condition: 5.

Stress relaxation measurements of these same hydrogels incubated cell media with no PEG after previous incubation in 10% (w/v) PEG at a range of PEG MW (0 kDa, 300 kDa, 600 kDa, 1,500 kDa, 3,000 kDa, 6,000 kDa, 8,000 kDa, and 35,000 kDa) are shown.

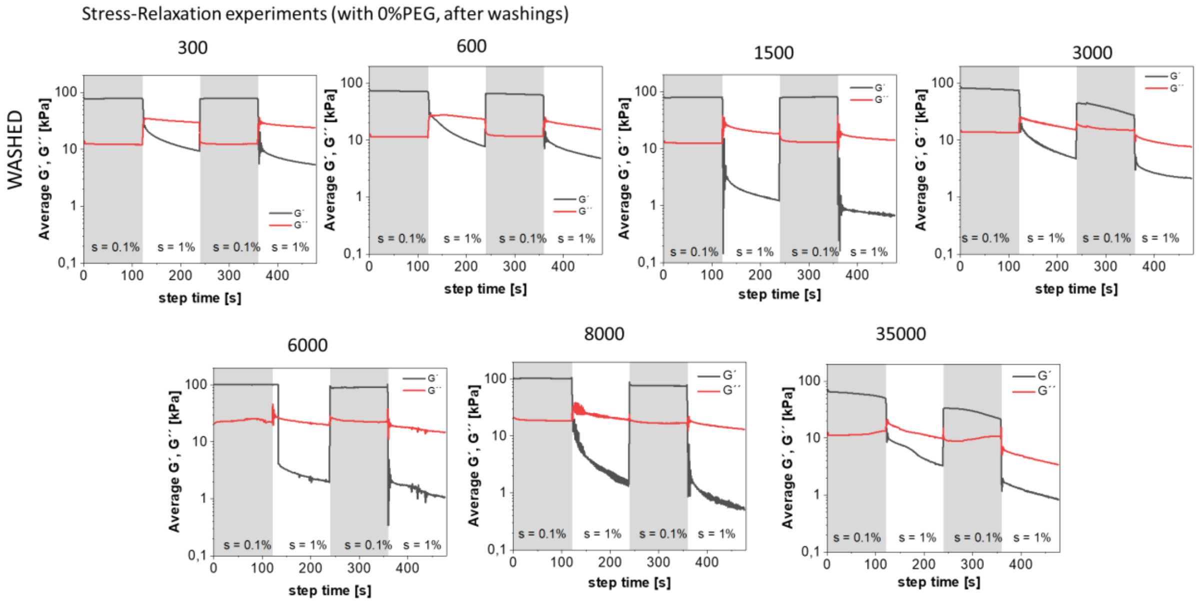

Figure S13. Rheological stress relaxation experiments of $CaCl_2$ cross-linked alginate gels incubated with 10% (w/v) PEG at a range of PEG molecular weights (MW) (0 kDa, 300 kDa, 600 kDa, 1,500 kDa, 3,000 kDa, 6,000 kDa, 8,000 kDa, and 35,000 kDa). Alginate hydrogels incubated with PEG are measured are measured at constant pH 7.5, 0.1% to 1% strain, 1 Hz frequency, and 37°C. Number of gels per condition: 5.

For comparison, the data of 2% alginate hydrogels incubated in 10% (w/v) PEG at a range of PEG MW in Fig. S12 and the same hydrogels after incubation in no PEG, as shown in Fig. S13, are presented in a single graph.

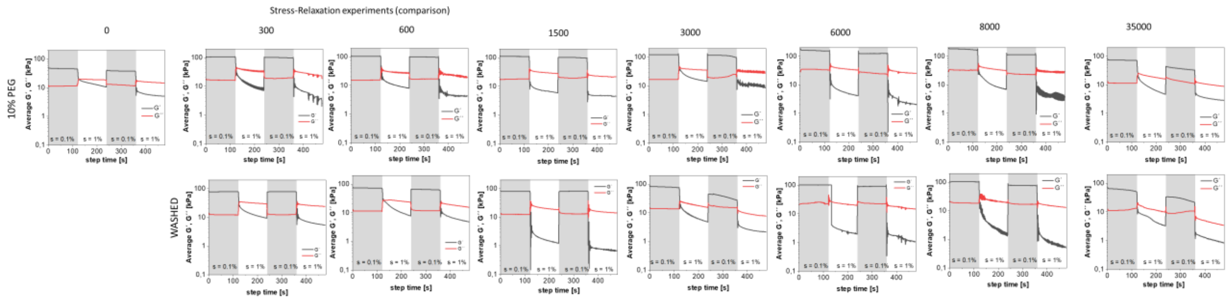

Figure S14. Rheological stress relaxation experiments of $CaCl_2$ cross-linked alginate gels incubated in (*above)* 10% (w/v) PEG at a range of PEG molecular weights (MW) (0 kDa, 300 kDa, 600 kDa, 1,500 kDa, 3,000 kDa, 6,000 kDa, 8,000 kDa, and 35,000 kDa) in comparison to these same alginate hydrogels (*below)* after incubation in cell media with no PEG. Washed alginate hydrogels are measured are measured at constant pH 7.5, 0.1% to 1% strain, 1 Hz frequency, and 37°C. Number of gels per condition: 5.

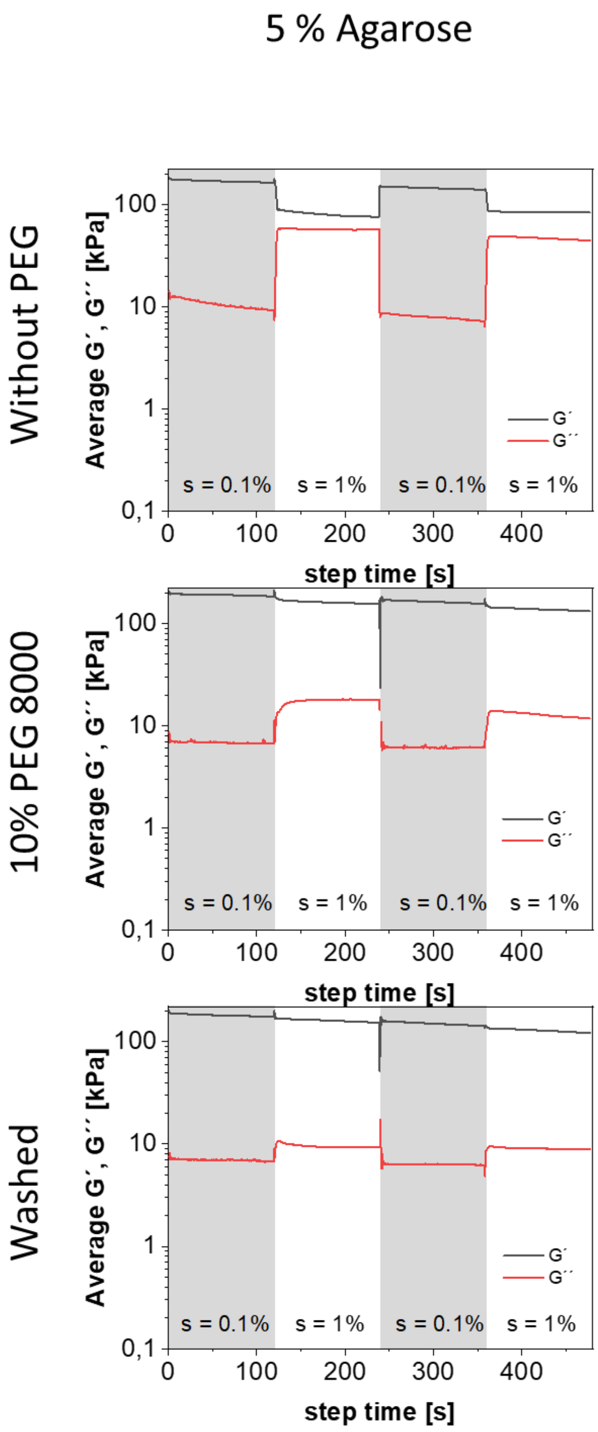

Figure S15. Rheological stress relaxation experiments of 5% agarose gels incubated with and without 10% (w/v) 8 kDa PEG. Agarose hydrogels with and without PEG incubation are measured at constant pH 7.5, 0.1% to 1% strain, 1 Hz frequency and 37°C. Washed agarose hydrogels are

those that were previously incubated in 10% (w/v) 8 kDa PEG, then incubated in cell media without the presence of PEG. Number of gels per condition: 5.

2. Rheological Comparison of Different PEG Concentrations

Rheometry measurements of 2% alginate incubated in different concentrations of 8 kDa PEG were performed, as in Fig. S10. Unlike the data presented in the main manuscript, the force applied to the alginate hydrogels was 0.5 N, which is half of the principal data, resulting in weaker measurements of G' and G''. Despite this difference, G' increases with 8 kDa PEG concentration, indicating that more interactions between the PEG and alginate polymers occurs due to the increasing number of PEG molecules.

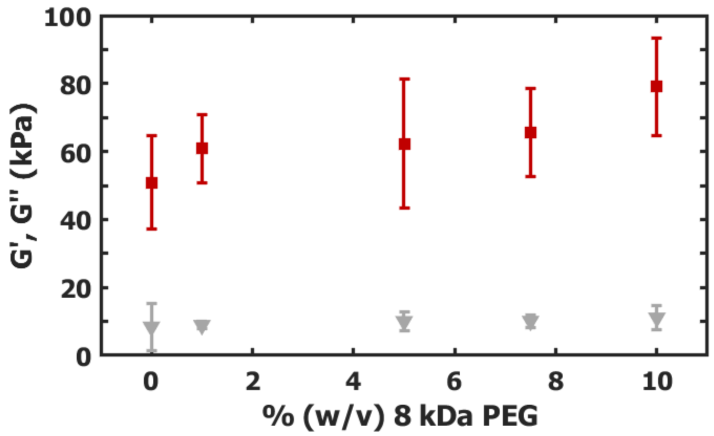

Figure S16. Rheological comparison of 2% alginate hydrogels incubated in different 8 kDa PEG concentrations (0% to 10% (w/v)). The hydrogels were measured at constant pH 7.5, 1 Hz frequency, 0.1% shear strain and 37°C. Number of gels for each condition: 6.

3. Rheological Comparison of Different Alginate Concentrations vs PEG Interacting Hydrogels

For comparison purposes, rheometry measurements of 2% alginate hydrogels incubated in solutions with 10% (w/v) 6 kDa and 8 kDa PEG are compared to 6% and 7% alginate hydrogels, as in **Fig. S17**. Higher alginate concentrations than 7% (w/v) alginate were not prepared as agarose at these concentrations did not dissolve in water at 90°C.

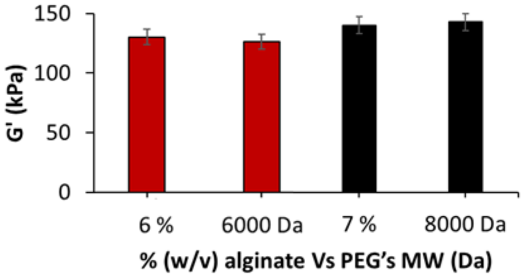

Figure S17. Rheological comparison of different alginate concentration (6% and 7% (w/v)) Vs 10% (w/v) (6 or 8 kDa) PEG interacting 2% (w/v) alginate gels, measured at constant pH 7.5, 1 Hz frequency, 0.1% shear strain and 37°C. Number of gels for each condition: 6

4. Solvent Effects on Hydrogels

Alginate hydrogels are polymeric in nature. Within a polymer hydrogel (or a polymer gel in general), the fraction of space occupied by polymers compared to its overall volume is defined by $\phi = V_{poly}/V_{total}$. This volume fraction is directly proportional to the hydrogel's polymer

concentration ($\phi \propto C$). According to rubber elasticity theory, the Young's modulus E of a pure polymer material is

$$E = 3\phi\upsilon_e N_A k_B T \tag{1}$$

where $\upsilon_e$is the solvent-free effective polymer chain density, N_A is Avogadro's number, k_B is Boltzmann's constant, and T is the temperature.[1] At constant temperature for a given material, both υ_e and T are constant; under these conditions, if the material swells or contracts, its volume fraction will decrease or increase, respectively, leading to a similar change in mechanical stiffness, E. Another common way of measuring polymer material mechanical properties is via the shear modulus G, related to Young's modulus via $G = \frac{E}{2(1+\nu_P)}$, where ν_P is Poisson's ratio, an inherent material property. G is thus also proportional to ϕ. 3

Alginate elastic moduli were calculated from rheometry data for comparison purposes with micro-indentation data, assuming *v* = 0.45–0.5, and are presented in **Fig. 2c**.

Table S1. Elastic Moduli for 2% (w/v) alginate without PEG and with 10% (w/v) 300 Da or 8 kDa PEG.

**Hydrogel**	**Elastic Modulus (kPa)**
2% Alginate	242±61

2% Alginate – 300 Da PEG	263±40
2% Alginate – 8000 Da PEG	455±130

In essence, the surface of a hydrogel works like a semi-permeable membrane between the hydrogel and the surrounding solution. As the hydrogel polymers are crosslinked together, the hydrogel itself acts as an aqueous solution mixed with concentrated solutes (the crosslinked polymers) incapable of leaving the semi-permeable membrane. When bathed in pure water, the hydrogel will swell to its maximum, decreasing ϕ and lowering G. If this swollen hydrogel is then transferred to a solution crowded with solutes, such as PEG, the difference in overall solute concentration, amongst others, will cause water to flow towards the more concentrated system. This pressure can be termed Π_{sol}, and will be a function of the PEG and other dissolved molecules. The overall pressure that either pushes solvent molecules into, or out of, the hydrogels is the sum of these two terms, $\Pi = \Pi_{sol} + G_e$. If left long enough, such a system will attain equilibrium, and $\Pi = 0$ with $G_e = -\Pi_{sol}$. If crowded solvents are used at a molar concentration lower than that of the hydrogel, a net outflux of solvent occurs, concentrating the hydrogel, increasing ϕ, and thus G.

5. Rheological Characterization of PEG-interacting and Standard Agarose Hydrogels

As with the alginate hydrogels, rheometry measurements of agarose hydrogels were performed using an 8 mm parallel plate geometry (HR 20 Discovery Hybrid Rheometer, TA Instruments, DE). A 5% (w/v) gel was prepared by weighing out the appropriate amount of agarose in a beaker and adding the appropriate amount of miliQ water to the flask containing agarose. The agarose-

water mixture was heated in the microwave until the agarose was completely dissolved, and left to cool. PEG-agarose gels were prepared by incubating individual hydrogels in solutions containing 8 kDa 10% (w/v) PEG (1 mL of PEG solution per 50 mg of agarose hydrogel). The gels were incubated for 10 days at 4 °C before measuring the mechanical properties to ensure an equilibrium concentration of PEG molecules both inside and outside the agarose network. An initial measurement of 1 N splitting force was set and experiments were performed at 1% shear strain, 1 Hz frequency and controlled temperature of 25 °C, as in **Fig. S18e**.

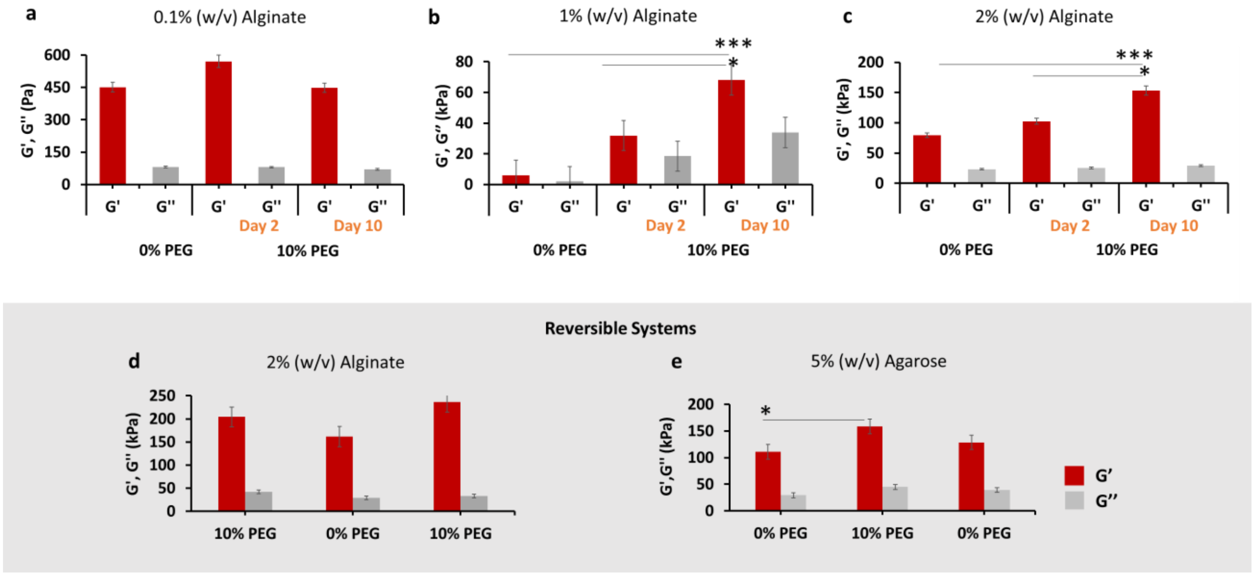

Figure S18. PEG-interaction effect on alginate or agarose hydrogels, for 10% (w/v) 8 kDa PEG on storage (G') and loss (G'') moduli, as measured by oscillatory rheology at constant pH 7.5, 1 Hz frequency, 0.1% shear strain, and 37°C. Interaction effects on 0.1% (**a**), 1% (**b**) and 2% (**c**) alginate hydrogels. Number of hydrogels for each condition: 8. (**d, e**) Effect of dynamically changing PEG concentration every 10 days on storage (G') and loss (G'') moduli for (**d**) 2% alginate hydrogels (**e**) 5% agarose hydrogels, as measured by oscillatory rheology at constant pH

7.5, 1 Hz frequency, 0.1% shear strain and 37°C. Number of hydrogels for each condition: (**d**) 5 and (**e**) 3.

6. Live/Dead Cell Assays

Live/dead cell assays were performed on cultured fibroblast cells under several conditions: for cells grown on the plastic well and not exposed to PEG (positive control), cells grown on the plastic well, then killed with 70% ethanol (negative control), and cells grown on 2% alginate and not exposed to PEG, as in **Fig. S19.**

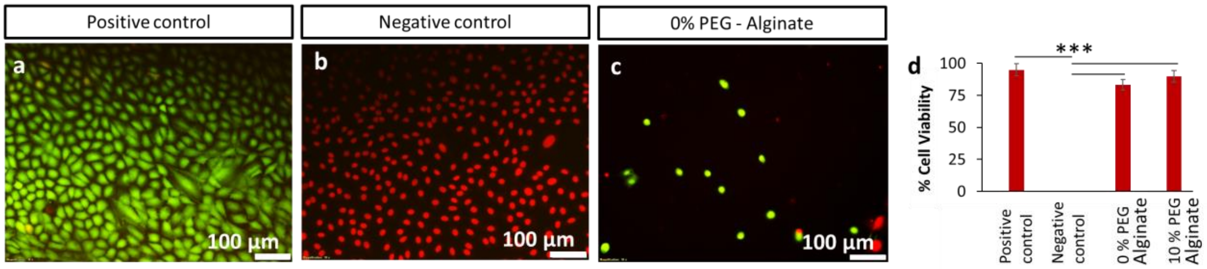

Figure S19. Live (green)/ dead (red) assay of 2D fibroblast cells cultured on alginate hydrogels under different conditions. Experimental conditions for cells: 2% (w/v) alginate with/without 10% (w/v) 8 kDa PEG in DMEM, 25 mM HEPES, pH 7.5, 37°C. **(a)** Positive control: untreated cells; **(b)** negative control: killed cells by ethanol; **(c)** alginate gels with 0% (w/v) PEG; **(d) % cell viability.** Scale bars: 100 µm. Number of experiments: Three, each with triplicates.

7. Quantification of Cells During Dynamic Tuning of Cell Adhesion and Expansion on the Alginate Hydrogels and Treated Well Plastic

By changing the PEG concentration, we can dynamically tune alginate hydrogel viscoelasticity (**Fig. S20b**) and thus dynamically affect cells grown/adhesion on these hydrogels (**Fig. S20a**).

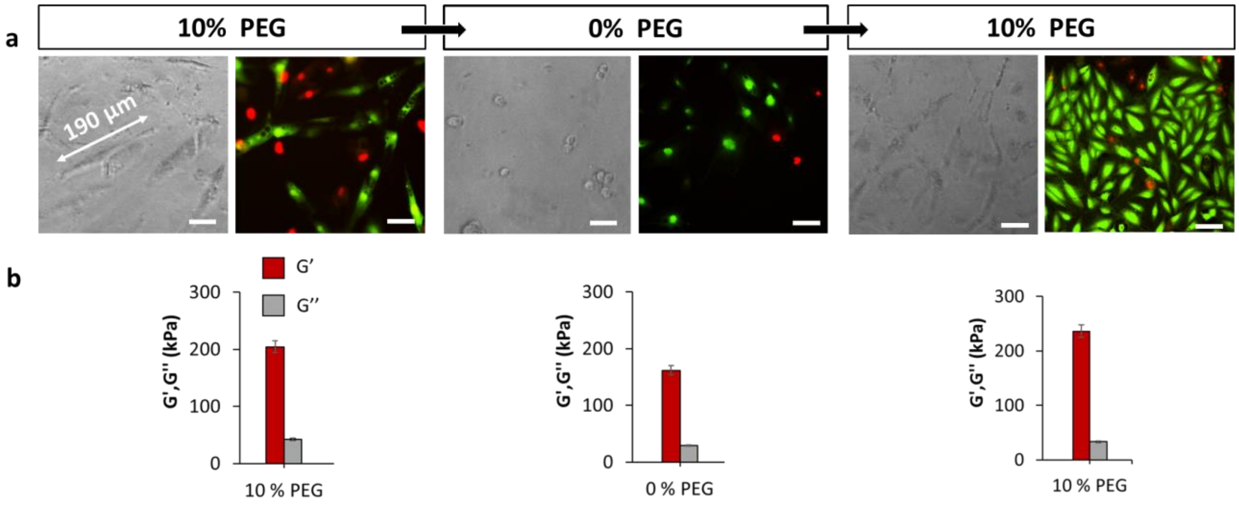

Figure S20. Dynamic Tuning of Cell Adhesion and Expansion on the Alginate Hydrogels. (a) Cell attachment to the alginate was controlled by modifying PEG concentration: from 10 % PEG to 0 % PEG (10 days without PEG) and back to 10 % PEG (10 days with PEG). Cell morphology changes from stretched, to globular, to stretched as the hydrogel viscoelasticity changes. **(b)** Effect of changing PEG's concentration (every 10 days) on storage (G') and loss (G") moduli of the alginate gels (2 % (w/v) alginate with/without 10% (w/v) 8 kDa PEG used for dynamic tuning), as measured by oscillatory rheology at constant pH 7.5, 1 Hz frequency, 0.1 % shear strain and 37°C. Number of experiments: Three, each with triplicates.

When PEG interactions are used to dynamically tune hydrogel viscoelasticity, cells demonstrate a stretched, spread-out morphology when compared to their round shape when grown on materials without PEG interactions (**Fig. S20a**). Cell morphology was further quantified over time, measuring the normalized number of cells (**Fig. S21a**), cell body length (**Fig. S21b**), and cell circularity (**Fig. S21c**) for cells grown on collagen-treated alginate hydrogels, exposed to PEG in

solution over 10 days, then measured again in media without PEG at 3 and 7 days. Additional controls were performed quantifying these same parameters for cells grown directly on the plastic well plate either: (i) with the plastic treated with collagen and exposed to PEG in cell media (PEG-DMEM-Col), (ii) on the plastic and exposed to PEG in cell media (PEG-DMEM), (iii) with the plastic treated with collagen and exposed to regular cell media (DMEM-Col), (iv) on the plastic and exposed to regular cell media (DMEM). Normalization was performed by scaling the total number of cells in each condition to that of cells grown on collagen-treated well plastic with regular DMEM cell media.

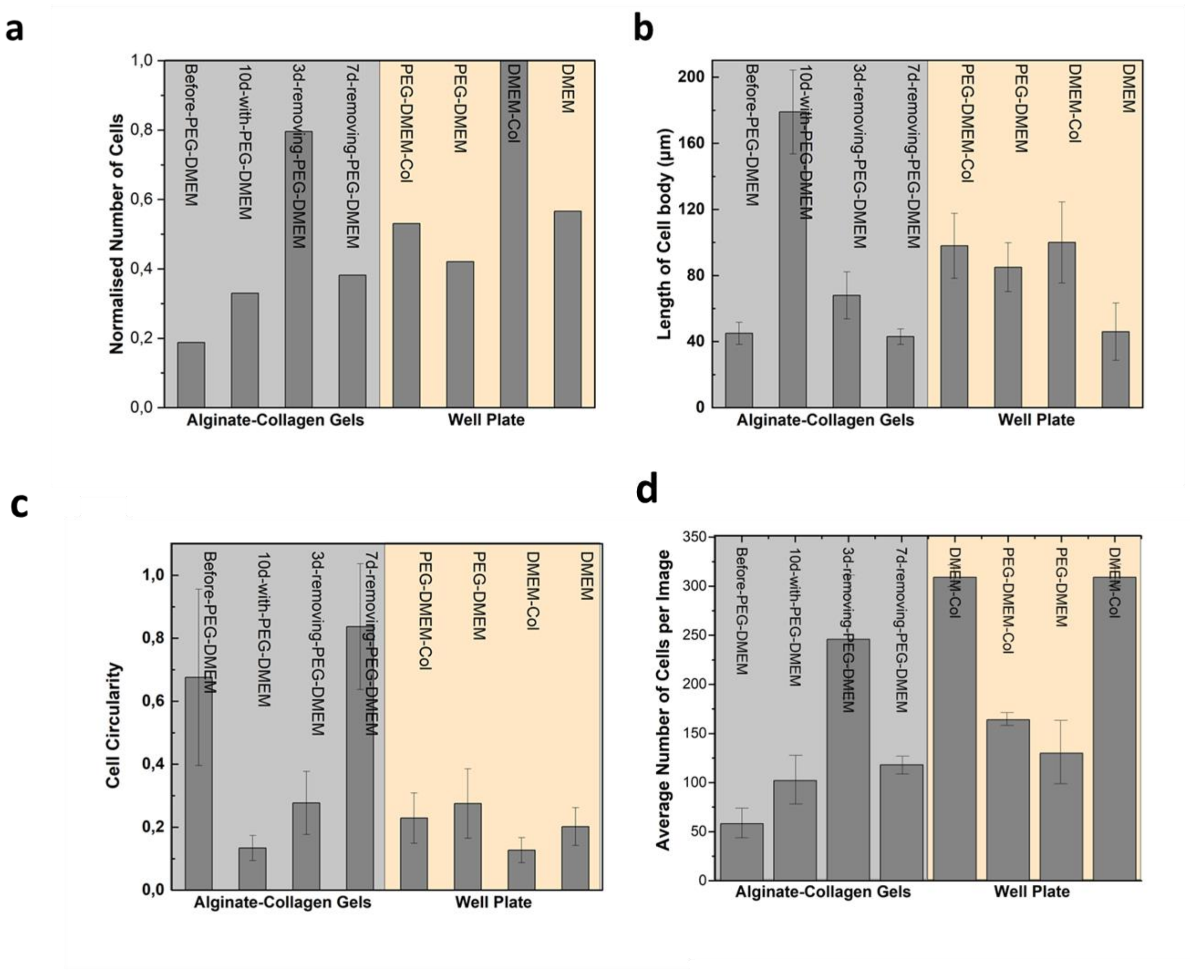

Figure S21. Dynamic Tuning of Cell Adhesion and Expansion on the Alginate Hydrogels and on Treated Well Plastic. To quantify the effects PEG-induced stiffening has on cell morphology, **(a)** the normalized number of cells, **(b)** length of the cell body, and **(c)** cell circularity as a function of time on collagen-treated alginate hydrogels are presented in gray. **(d)** Average number of cells per image. Controls of cells using various treatments of the well plastic are presented in yellow. Number of experiments: Three, each with triplicates. Number of measured images: 8. Number of cells per image (between 15 – 45 cells).

Figure S21a demonstrates that PEG present in solution may inhibit cell growth, with more cell growth on the collagen-treated well plate. However, cells remain healthy enough that even after 10 days of growth in PEG-supplemented media, replacing this media with regular DMEM lacking PEG allows the cells to grow exponentially. Additionally, while cell morphology was strongly affected by the presence of PEG in solution when cells were grown on hydrogels, this is likely due to the stiffening effect caused by PEG interacting with the hydrogel: Cells exposed to PEG on well plates have similar morphological traits to cells grown on collagen-treated well plates with no PEG exposure.

To further investigate the effect of PEG in media on cell viability, we observed the number of cells per unit area (in cm^2) initially without PEG, several days after the addition of PEG, then again after PEG removal (Figure S22). Here, cell viability was investigated using a cell population from the same well as opposed to comparing them to the "ideal" situation where cells were grown directly on collagen-treated plastic and only exposed to DMEM without PEG. Cell density increased after several days' growth in the presence of 10% (w/v) 8 kDa PEG compared to growth in regular DMEM media with no PEG, indicating that the addition of PEG did not significantly hamper cell viability. Additionally, after changing the media to DMEM without PEG and allowing cells to continue to grow for several days, cell density decreased. This may be due to the cell density reaching a very high level on a day where cell density was not measured, leading the cell population to enter apoptosis (pre-programmed cell death) and thus a decrease in cell density.

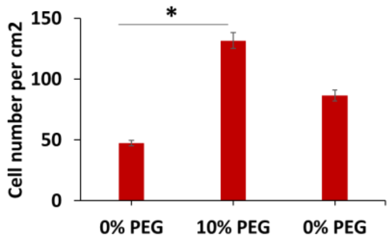

Figure S22. Cell Population Observed While Varying the Presence of PEG. Cells were grown on 2% alginate hydrogels and exposed to regular cell media, with the number per unit area in several wells counted. The cell media was then exchanged with media mixed with 10% (w/v) 8 kDa PEG, and the number of cells per unit area was counted after being left to grow for several more days in this PEG-supplemented media. Finally, this media was exchanged with regular cell media, and the cell number per unit area was quantified after several days' growth. Number of experiments: Three, each with triplicates. Number of measured images: 8. Number of cells per image (between 15 – 45 cells).

8. Cells Grown on High Stiffness Alginate Hydrogels with No PEG vs Those Grown in the Presence of PEG

To verify that the observed morphological changes in the cells were exclusively due to changes in substrate stiffness induced by polyethylene glycol (PEG) interactions and not influenced by metabolic factors, crowding effects of PEG in solution or interactions between PEG and the collagen used to coat the hydrogels, cells were cultured on 7% (w/v) alginate hydrogels without exposure to PEG. These cells were then incubated with DAPI, phalloidin and paxillin for staining of cellular components and microscopic analysis was performed as described in the main text (**Fig. S23**).

At the indicated concentration, the alginate hydrogels exhibited a storage modulus (G') comparable to that of 2% (w/v) alginate incubated in 10% (w/v) 8 kDa PEG, with the 7% (w/v) alginate exhibiting only a slightly reduced stiffness (**Fig. S23**). This similarity is evident in the nearly identical cell body lengths of cells cultured on the 7% (w/v) alginate compared to those on the 2% (w/v) alginate incubated in 10% (w/v) 8 kDa PEG, with the former showing only a slight reduction

in body length (**Fig. S23**). This slight difference in body length can be attributed to the slightly lower G' of the 7% alginate compared to the 2% alginate incubated in 10% (w/v) 8 kDa PEG. Furthermore, these results suggest that the observed changes in cell morphology are not due to interactions between the PEG in solution and the collagen coating on the hydrogels. In such a scenario, cells would respond exclusively to the 2% (w/v) alginate hydrogel incubated in PEG, rather than showing similar responses to the 7% (w/v) alginate that remained unexposed to PEG. Thus, these results confirm that differences in cell morphology within samples cultured on 2% alginate incubated with 10% (w/v) 8 kDa PEG are solely due to changes in the rigidity of the alginate and are independent of any direct effects of PEG on the cells.

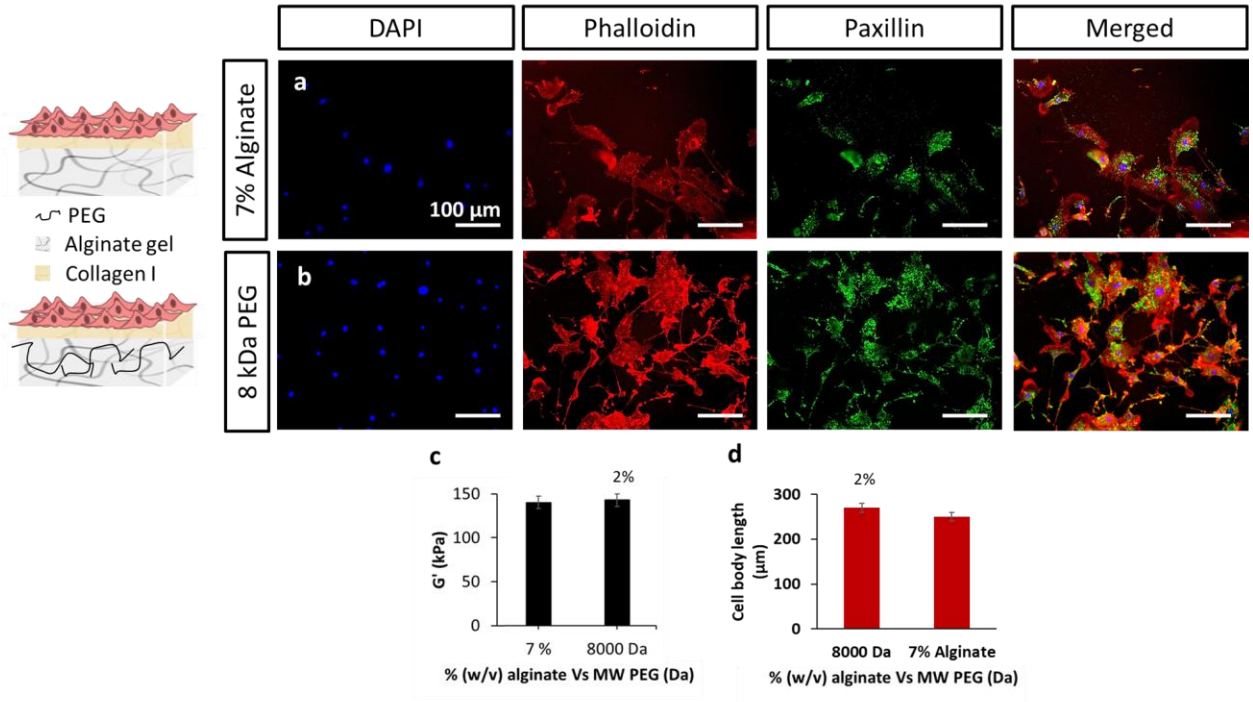

Figure S23. Cells Grown on Alginate of a Similar Stiffness to Those Incubated with PEG Show Little Difference in Cell Morphology. Cells were grown on both **(a)** 7% alginate with no exposure to PEG, and **(b)** on 2% alginate with 10% (w/v) 8 kDa PEG incubation, then stained with DAPI, phalloidin, and paxillin. Cells demonstrated similar morphologies, regardless of the alginate

hydrogels they were grown upon. **(c)** The hydrogels' G' were also measured, with that of the 7% alginate being only slightly lower than that of the 2% alginate incubated in 10% (w/v) 8 kDa PEG. This translated to a practically indistinguishable difference (very slightly smaller) body length for cells grown on the 7% alginate than those grown on the PEG interacting 2% alginate using 10% (w/v) 8 kDa PEG. These results indicated any cell morphology differences are due to the difference in hydrogel G', and not any other effect of PEG on the cells. Number of experiments: Three, each with triplicates. Number of cells per image (between 20 – 35 cells).

9. HPLC Calibration Curve

To verify the incorporation of PEG into the alginate gels, samples of a fluorescent PEG (FITC-labelled or FITC-PEG) solution used for incubation were measured by addition to an RP-HPLC before and after incubation of alginate hydrogels in the FITC-PEG solution for 10 days. For this experiment, 100 µL of 10% (w/v) 8 kDa FITC-PEG in water was diluted with 900 µL of deionised water (DIH_2O, 1000 µL total) and then injected into the HPLC before addition to the hydrogels. A peak in absorbance intensity is observed after 15 minutes (**Fig. S24**). After exposing the hydrogels to 10% (w/v) 8 kDa FITC-PEG for 10 days, 100 µL of the FITC-PEG solution surrounding the hydrogels was again diluted in 900 µL DIH_2O (1000 µL total) and injected onto the RP-HPLC column. This solution showed a much smaller peak after 15 minutes, indicating a reduced concentration of FITC-PEG, probably due to penetration of the FITC-PEG into the alginate hydrogel (**Fig. S17**). For comparison, DIH_2O was also injected into the RP-HPLC and no peak was observed at 15 minutes. As the injected concentration was the same, we quantified the change in area under the curve by taking 100% of the PEG spot (without contact with hydrogels) and calculating the percentage of the other smaller spots. The consumption of 30-40% of the initial

PEG solution was confirmed by RP-HPLC after 10 days of incubation in contact with the hydrogels.

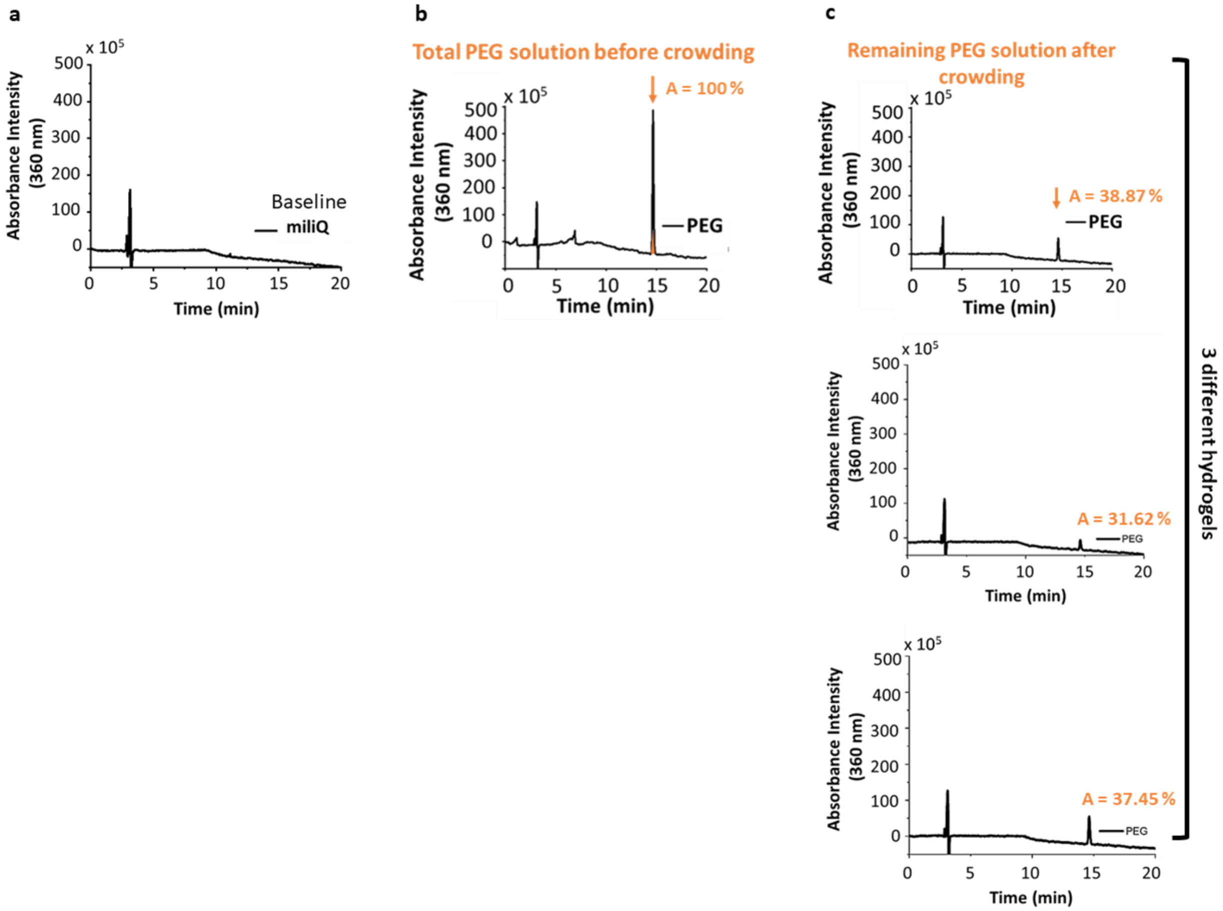

Figure S24. Absorbance spectra for Solutions Tested in RP-HPLC. To test the uptake of fluorescent PEG (FITC-PEG) into alginate hydrogels, 100 µL of a 10% (w/v) 8 kDa FITC-PEG was diluted in 900 µL of DIH2O and injected into an RP-HPLC. A peak is visible at 15 minutes, attributed to FITC-PEG coming off of the column. After incubating a 2% alginate hydrogel in the FITC-PEG solution for 10 days, another 100 µL of the 10% (w/v) 8 kDa FITC-PEG used to incubate the hydrogel was diluted in 900 µL of DIH2O and injected into the RP-HPLC. Another smaller peak was observed at 15 minutes, indicating a significant quantity of FITC-PEG was lost

through diffusion into the alginate hydrogel. A control experiment using DIH2O was injected into the RP-HPLC, with no peak at 15 minutes occurring. Number of experiments: Three gels in total.

10. Cells Grown on Fibronectin-functionalized vs Collagen-functionalized Alginate

As fibronectin is commonly used as a cell-adhesive protein, we tested whether fibronectin or type I collagen would be best suited for our cell experiments in the presence of PEG. Fluorescence microscopy of the cells grown on collagen-functionalized vs. fibronectin-functionalized substrates demonstrate lower cell proliferation on fibronectin-functionalized hydrogels. This was true regardless of whether the hydrogels were incubated in either 0% PEG and 10% (w/v) 8 kDa PEG, as can be seen in **Fig. S25**. This difference between the two different protein coatings for cell growth may arise due to the much higher crosslinking efficiency of collagen with EDC than for fibronectin with EDC. As such, type I collagen was used to functionalize our alginate hydrogels for all experiments.

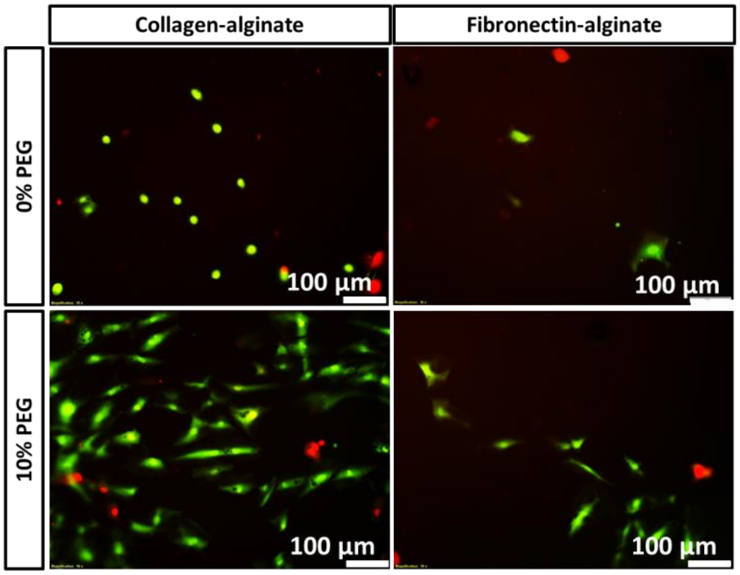

Figure S25. Live (green)/ dead (red) assay of fibroblast cells cultured on either type I collagen-functionalized or fibronectin-functionalized alginate hydrogels. Cells were grown either in media with 0% PEG, or 10% (w/v) 8 kDa PEG for several days. Cell proliferation was superior on alginate hydrogels functionalized with type I collagen than on those functionalized with fibronectin**.** Scale bars: 100 µm. Number of independent experiments, with dublicates: 3

References

[1]. Sato, K. *et al.* Phase-Separation-Induced Anomalous Stiffening, Toughening, and Self-Healing of Polyacrylamide Gels. *Adv. Mater.* **27**, 6990–6998 (2015).
